# Epithelial cancers in the post-genomic era: should we reconsider our lifestyle?

**DOI:** 10.1007/s10555-013-9445-5

**Published:** 2013-08-02

**Authors:** Jeff M. P. Holly, Li Zeng, Claire M. Perks

**Affiliations:** School of Clinical Science, Faculty of Medicine, University of Bristol, Learning and Research Building, Southmead Hospital, Bristol, BS10 5NB UK

**Keywords:** Metabolism, Breast cancer, Prostate cancer, Colorectal cancer, Lifestyle

## Abstract

The age-related epithelial cancers of the breast, colorectum and prostate are the most prevalent and are increasing in our aging populations. Epithelial cells turnover rapidly and mutations naturally accumulate throughout life. Most epithelial cancers arise from this normal mutation rate. All elderly individuals will harbour many cells with the requisite mutations and most will develop occult neoplastic lesions. Although essential for initiation, these mutations are not sufficient for the progression of cancer to a life-threatening disease. This progression appears to be dependent on context: the tissue ecosystem within individuals and lifestyle exposures across populations of individuals. Together, this implies that the seeds may be plentiful but they only germinate in the right soil. The incidence of these cancers is much lower in Eastern countries but is increasing with Westernisation and increases more acutely in migrants to the West. A Western lifestyle is strongly associated with perturbed metabolism, as evidenced by the epidemics of obesity and diabetes: this may also provide the setting enabling the progression of epithelial cancers. Epidemiology has indicated that metabolic biomarkers are prospectively associated with cancer incidence and prognosis. Furthermore, within cancer research, there has been a rediscovery that a switch in cell metabolism is critical for cancer progression but this is set within the metabolic status of the host. The seed may only germinate if the soil is fertile. This perspective brings together the different avenues of investigation implicating the role that metabolism may play within the context of post-genomic concepts of cancer.

## Introduction

In the developed world, malnutrition and infectious diseases no longer restrict lifespan resulting in a shift in the demographics with increasing populations of elderly people and increasing prevalence of age-associated disorders. Cancer is one such age-associated disorder and is an ever increasing challenge for Westernised societies. In the USA, cancer is the number two killer with around 1.5 million new cases each year and more than a half million deaths. It is also now the main cause of premature death, having overtaken cardiovascular disease as the major cause of mortality in all but those over the age of 85 years [1]. For most people, a premature death is of greater concern than death into the eighth or ninth decade after a full life. In 1971, President Richard Nixon famously declared war on cancer with the mission: ‘to find a cure for cancer’. In the subsequent four decades, there have been many major advances in our understanding of the molecular pathology of cancer but these advances have been slow to translate and to have major impact in the clinic. In the USA, during the period 1951–2001, the death rates from heart disease, cerebrovascular disease, pneumonia, and influenza were reduced by more than half but in contrast the death rate from cancer remained unchanged [2].

For many decades, the favoured concept for why we get cancer has been the somatic mutation theory, with cancer being caused by an accumulation of gene mutations: either from gain of function of oncogenes or from loss of function of tumour suppressor genes or mutations in DNA-integrity genes [3]. Research identifying and characterising these cancer genes was so successful that the concept that these gene mutations were the most important determinants of cancer became the widely accepted dogma. This created early hope that studies of oncogenes and tumour suppressor genes would yield many new treatment targets resulting in numerous, novel interventions that would revolutionise how we manage clinical cancers. Although our knowledge and understanding has grown enormously over the last few decades, much of that early hope has not been fulfilled. The exploration of the cancer cell genome has led to dramatic developments in the treatment of some cancers, such as childhood leukemia, Hodgkin’s disease and testicular cancer. In addition, the overall mortality from some of the common cancers has been falling recently because of advances in early detection and treatment. The statistics may also be distorted because of the increasing detection of early indolent cancers that would never progress to fatal disease leading to a false impression of increasing survival. Despite these advances, for the most common epithelial cancers (lung, breast, prostate and colorectal), the prognosis for patients with metastatic disease has seen little improvement. The vast majority of deaths due to cancer result from metastatic disease that is resistant to current therapies [4]. There has however been an accumulation of evidence from a variety of fields that when integrated together may give us a clearer view of these cancers and the clinical challenges that they present.

## Epithelial cancers have large numbers of mutations but generally these are naturally acquired

Despite popular perception, cancers generally do not arise from a mutation in a single gene or even from mutations in a small number of genes. Elaborate mechanisms have evolved to protect against gene mutations directly leading to lethal cancers. It is now clear that by the time a tumour is clinically apparent, it has accumulated a large number of mutations, many of these are not within gene coding sequences (the significance of which is yet to be understood) and there is considerable variation in mutations between tumours even those in the same tissue. Mutations contributing to cancer pathophysiology are termed driver mutations whereas those not currently known to contribute are termed passenger mutations. It is thought that for most cancers the initiation and progression is primarily due to a few common driver mutations but there are also many additional plausible driver mutations that occur at low frequency or are idiosyncratic to an individual clone of cells. The malignant phenotype is a result of the collaborative effect of these many mutations that could number into their thousands for any particular tumour. For breast and colorectal cancers, one study estimated that there were around 100 mutations per cancer that were nonsynonymous (affecting coding for protein sequence) with as many as 20 suggested as driver mutations potentially playing a role in carcinogenesis [5, 6].

Gene mutations occur generally because of errors during DNA replication or from exposure to genotoxic agents. Cells that are frequently replicating are at much higher risk and although cell proliferation is most active during early development and childhood, in general human cancer is a disease of aging: presumed to be due to the requirement for an accumulation of mutations. Errors in DNA replication occur naturally at a rate of around 10^−10^ to 10^−9^ per nucleotide per cell per division: this is not a failure in the biology but indeed is central to evolution [7]. It now seems likely that most cancers are initiated in cells because of this normal mutation rate: studies that have measured the numbers of mutations in common cancers have provided evidence consistent with this natural rate. Mutations that contribute to oncogenesis can occur in the germline and result in hereditary predisposition to cancer or more often they occur in single somatic cells that result in sporadic cancers. The vast majority (around 98 %) of human cancers that present clinically are sporadic, rather than inherited, and the majority of these (around 90 %) are carcinomas that generally occur in epithelial cells: these are cells that continuously divide to replenish epithelial barriers; for example, the human colon is a tissue with relatively high rates of epithelial cell division and has approximately 1–1.5 × 10^7^ crypts, each one maintained by a small number of stem cells. In mice the identification of a potential stem cell marker has enabled an estimation of crypt stem cell division as around once per day [8]. In humans, there is no such evidence but estimates of crypt stem cell division are between once every 1–4 days. Simple mathematics indicates that this should generate an accumulation of mutations over a life-course such that by the age of 70 years: a cell division rate of once per day would yield around 2.6 mutations/100,000 bases and a cell division rate of once per 4 days would yield 0.65 mutations/100,000 bases [9]. For colorectal cancers, actual measurements of the prevalence of mutations in tumours have now been reported as less than 1 mutation/100,000 bases [5, 10]. Combining these strands of evidence indicates that the normal mutation rate and the cell turnover rate are sufficient to generate cells with a cancer genotype over a normal lifespan. An important consideration is that these simple calculations are based on average rates calculated for the average cell, but in any individual human, a cancer is presumed to develop when just one cell accumulates the critical number and combination of mutations. The risk of just one, out of more than 15 million, crypt stem cells reaching this threshold is considerably greater than the risk for an average cell. The accumulation of genetic mutations in a tumour generates a cancer genome that varies from that of the somatic germline genome by <1 base/100,000 bases: this is a relatively small difference in comparison to the variance that occurs for the germline genome between different individuals. There is also huge variation in cancer genomes between patients even with tumours in the same tissue [5, 6]; this undoubtedly is partly due to the tumour genome variations developing from germline genomes that themselves vary considerably between individuals.

That a cancer genome can be generated naturally from normal cell division is also consistent with measures of the accumulation of mutations in normal tissues from individuals without cancer. A study of mutations in the oncogene p53 in normal cancer-free adults found that there were around 50 clones of cells with mutated p53 within every square centimeter of normal skin epithelium, each clone containing between 60 and 30,000 cells [11]. Another study examined the rate of somatic mutations at a single gene locus in kidney epithelial cells obtained from organ donors. In donors from the first decade of life, the rate of mutations observed was around 0.2 in 100,000 and there was an increase with age such that for donors in their eighth and later decades the rate was 4 in 100,000 [12]. In addition to the accumulation of mutations that occurs with age, there is also an increase in epigenetic modifications in normal tissues that can then also predispose to malignant transformation, for example, by the inactivation of tumour suppressor genes. In the prostate glands from men without cancer it has been observed that the methylation of promoter CpG islands in genes implicated in prostate cancer increased with age [13]. A recent study of clonal mosaicism, defined as large structural genomic events resulting in alteration of copy number or loss of heterozygocity, in a cohort of 31,717 people with cancer and 26,136 cancer free controls found that in the latter the strongest predictor of clonal mosaicism was age with a frequency of 0.23 % in subjects younger than 50 years increasing to 1.91 % in subjects aged 75–79 years with only a slightly higher frequency found in individuals with cancer and no increased frequency in those with early onset cancers [14].

**Fig. 1:**
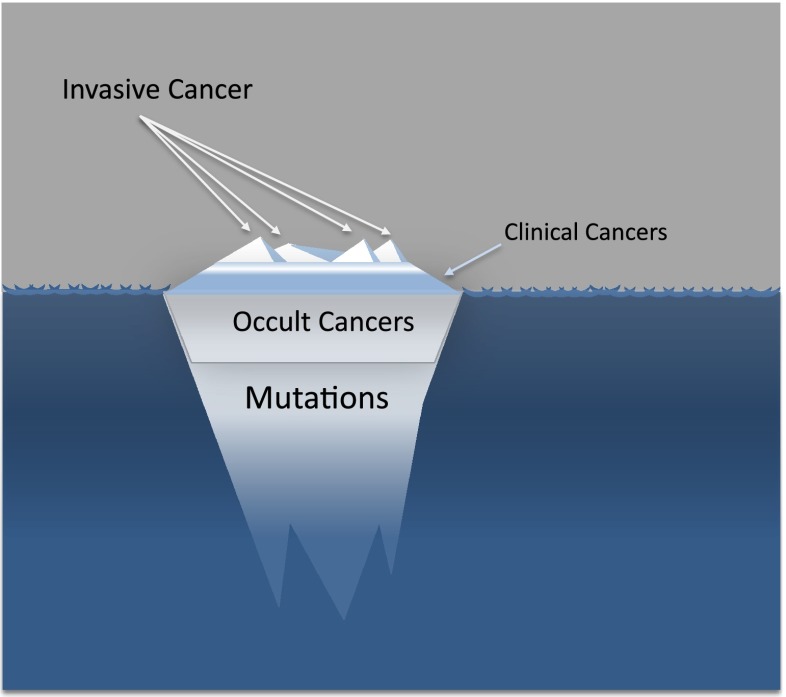
Epithelial cells in tissues with relatively high cell turnover accrue a large number of mutations by the time individuals reach their 6th or 7th decade. Elderly subjects harbour many epithelial cells with the number of mutations sufficient to induce malignant transformation. Most elderly individuals will as a consequence develop latent, occult neoplastic growths. In a normal lifespan, relatively few of these growths develop sufficiently to become clinically apparent cancers, and even fewer progress to invasive disease: the tip of a very large ‘iceberg’ of hidden cells with a malignant genome

Together, this evidence indicates that genetic aberrations, even major changes, accumulate normally as we age and the rates at which mutations develop in advanced cancers is very similar to that in normal cells [15] and hence a cancer genome may be acquired naturally with advancing age. With around 10^13^ cells in the body and many millions of cells normally acquiring the number of mutations sufficient to generate a malignant genotype by the age of 70, the inevitable conclusion is that in all individuals who survive into old age, there will always be plentiful cells with the requisite mutations and hence capacity to initiate a cancer. The incidence of clinical cancers is therefore relatively very low compared with the burden of potential cancer-causing mutations (Fig. 1).

## Cancer heterogeneity: more than genetic!

**Fig. 2:**
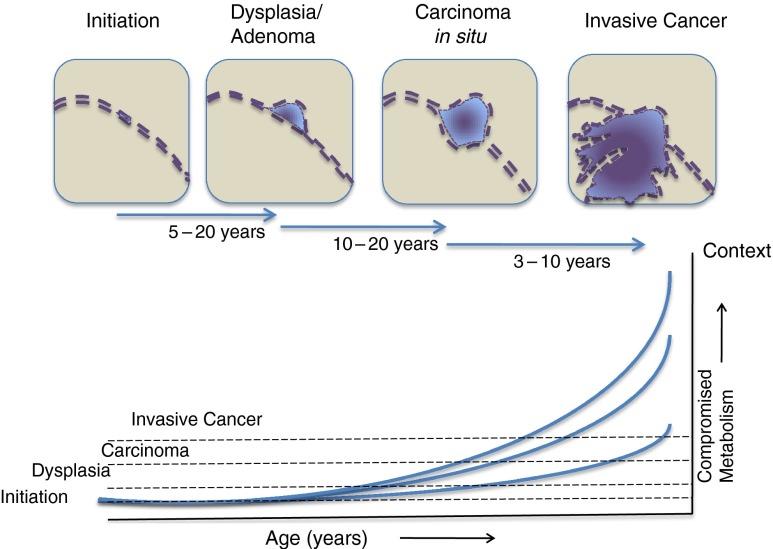
For most of the common epithelial cancers, there are decades between the occurrence of the initiating mutations and the appearance of a carcinoma. For individuals in their 60s or 70s, there are age-related declines in hormones reducing the drive for the progression of the neoplastic lesions that occur naturally with aging. The progression of these occult cancers is sufficiently slow such that within the normal lifespan of most subjects clinical disease does not have time to develop. With lifestyle-induced disturbances to metabolic control and the consequent hormonal imbalance, these occult tumours receive signals that the host organism is in a ‘fed state’ and in the transformed cells this can activate developmental programs promoting progression. In addition, these malignant cells are provided with a fertile soil in which to develop. The process can then be accelerated such that more of the occult cancers progress to develop clinical disease

In addition to the considerable variation between tumours (intertumoural heterogeneity) in different individuals, even those of similar phenotype in the same tissue: it has gradually become clear that there can also be considerable heterogeneity within individual tumours and heterogeneity between the primary tumour and metastatic lesions within any cancer patient. This intratumoural heterogeneity can arise through a number of mechanisms. The first source is genetic heterogeneity: as the cancer cells pass through many rounds of division the acquisition of further mutations results in the development of clonal diversity. There then appears to be clonal selection according to Darwinian principles with clones selected that have preferred advantages to survive and grow within the local conditions and environment. The current limitation of most imaging techniques used to detect cancers is around 1 cm which equates to approximately 10^−9^ cells, which when considered with the average tumour doubling-time, provides an estimate of around 12 years between cancer initiation and diagnosis [4]. This represents at least 30 cell doublings with the potential accumulation of significant genetic heterogeneity. Studies on patients with colorectal cancer further indicate that the transition from an adenoma to a carcinoma may take 10–20 years [15] in which time genetic heterogeneity can further increase (Fig. 2). A sequencing analysis of multiple samples taken from four patients with renal-cell carcinomas, both before and after treatment, revealed considerable intratumoural heterogeneity [16]. A single tumour biopsy specimen revealed only a minority of the genetic aberrations in the entire cancer: 63 to 69 % of all somatic mutations were heterogeneous and not found in every sequenced region and between 25 and 50 % of nonsynonymous variants identified in single biopsies were private and not found elsewere in the cancer. Similar intratumoural heterogeneity has also been reported for breast cancer where even a single biopsy specimen was found to contain multiple intermixed karyotypic tumour populations [17]. Evidence also indicates that metastatic lesions (which eventually result in death) may be derived from a low frequency subclone that was present in the original tumour [18] which may therefore not be detected in an original biopsy.

The emerging evidence therefore indicates that concepts of tailoring personalised medicine and biomarkers according to an individual’s cancer genome established from a single-needle biopsy are extremely naïve. The evidence of intratumoural heterogeneity suggests that most individuals with epithelial cancers will probably have tens or hundreds of cancer ‘genomes’ and the genome most evident in an initial biopsy may not be the one that eventually threatens the patient’s life.

**Fig. 3:**
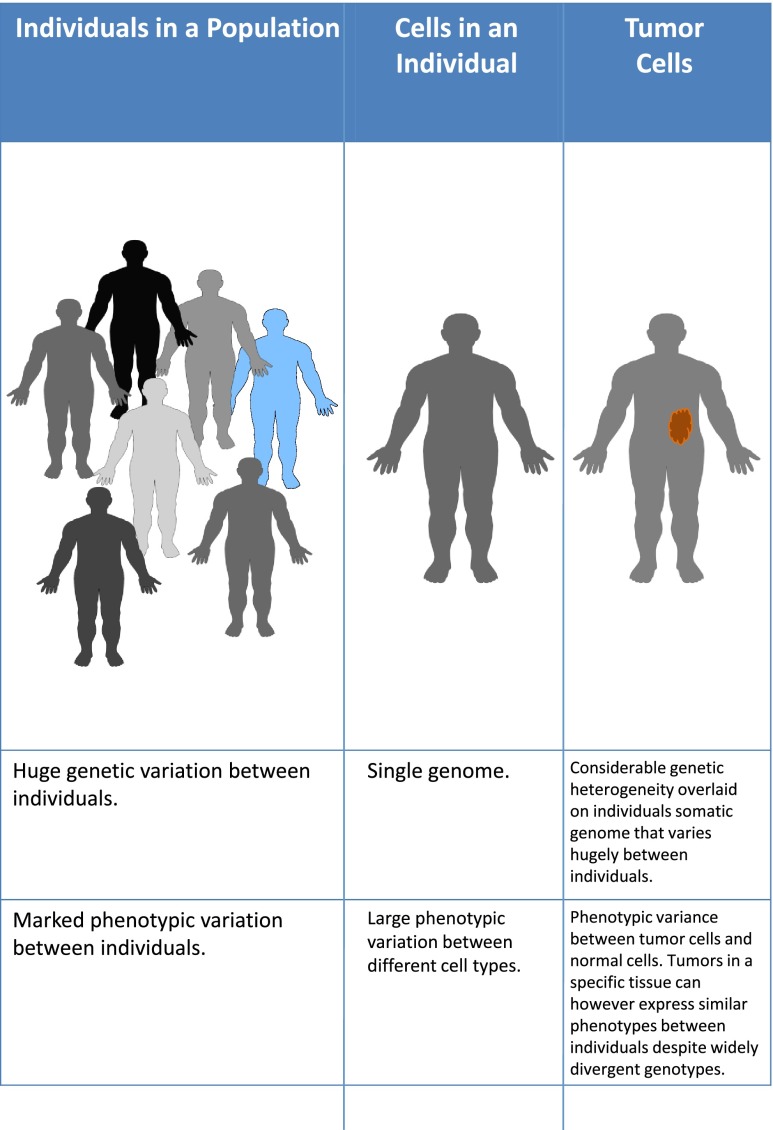
Within populations of humans there is considerable genetic heterogeneity between individuals. Within every individual, all of the somatic cells have an identical single genome but the cells develop considerable phenotypic heterogeneity: cells of the bone, skin, liver, nervous system, and immune system can have extremely different appearance and functions despite all being genetically identical. Within a tumour, the cells can acquire considerable genetic heterogeneity and a breast, prostate, or colorectal tumour in one patient can be genetically very different from that in another patient, partly because of the underlying somatic variations between individuals. Despite this considerable genetic diversity, tumours in the same tissue can manifest similar phenotypes

Currently, the most widely held view is that cancer progression occurs through multiple steps of acquiring further genetic changes and clonal selection of those with an advantage [19]. There is however a major paradox with this theory. The genes that may be selected to confer a growth advantage within the environment of the primary tumour site may not necessarily also confer an ability for the cell to be disseminated to a second, metastatic site where it may enter a long period of dormancy before again starting to proliferate. It has long been recognised that primary prostate cancers are generally multifocal composed of multiple genetically distinct cell clones. A study of multiple metastatic sites from men who had died of prostate cancer revealed that most metastatic lesions appeared to originate from a monoclonal source but the individual lesions then accumulated a variable number of subclonal changes [20]. This suggested that disparate metastatic deposits may have originated from a single subclone in the primary prostate cancer. There is also evidence that the metastatic genotype may be acquired early during tumourigenesis and then other sources of heterogeneity may drive progression [15, 21]. Indeed there are numerous normal mechanisms that enable considerable heterogeneity to develop from a single genotype. Every human develops from a single fertilised cell to become a complex organism comprised of billions of cells that can express vastly different properties and functions: cells such as keratinocytes, bone cells, lymphocytes and neurons are extremely different from each other and yet they all possess exactly the same single genome. Everyone accepts that this incredible heterogeneity of phenotypes can develop from a single genotype. It then seems incongruous that when considering why a breast cancer cell has a difference in phenotype from a normal breast epithelial cell (differences that in comparison are relatively small) explanations seem to require solely a genetic basis and a series of mutations are sought to explain every change in phenotype that is required to acquire the hallmarks of a cancer cell (Fig. 3).

A second potential source of intratumoural heterogeneity is now recognised to be due to epigenetic changes and adaptations. Acquired epimutations may in effect be like genetic mutations in silencing tumour-suppressor genes, however epigenetic changes may be reversible. As discussed later, some epigenetic changes may also be heritable and some may be affected by exposures. A third source of intratumoural heterogeneity comes from cell differentiation heirarchies. In an update of the embryonic theory of cancer, that suggested cancers arise from embryonic cells that are preserved in adult tissue, it has recently been popular to think that tumours have rare cells that retain stem cell like properties that can divide asymmetrically and retain a capacity for self-renewal. Recent evidence suggests that it is not so ‘black and white’ but that there are a spectrum of stable states that a cancer cell can adopt according to epigenetic and phenotypic stable conditions which the cell can flip between [22]. In a study of genetically homogeneous clones of human colorectal cancer cells, over multiple serial transplantations in mouse xenografts, it was found that there was considerable heterogeneity between the ‘genetically identical’ clones in terms of proliferation, persistence and chemotherapy tolerance. Furthermore these individual clones changed with exposures: following chemotherapy dominant clones emerged that were previously minor or dormant lineages [23].

It has also recently emerged that there is yet another source of heterogeneity because of stochasticity in the pattern of gene expression and activity of signalling pathways within cells [24]. In a traditional experiment, a collection of cells are exposed to a stimulus or stress and then after a given time the cells are then collectively broken up to extract mRNA and proteins which are then analysed to assess changes in gene expression or changes in activity of signalling proteins. Such an experiment however only gives a snapshot with a population-averaged and time-averaged result. However, not all the cells may be the same in that population, and they may not all be in the same phase of their response, but techniques limited our ability to obtain anything other than a population- and time-average response. In recent years, techniques for single cell analysis have enabled assessment of the natural heterogeneity between seemingly identical cells [25]. An elegant such study fluorescently tagged nearly 1,000 different endogenous proteins each in an individual, but genetically identical, cancer cell and then used time-lapse microscopy to follow how the individual cells respond over time to a common chemotherapy drug (camptothecin). All cells showed rapid translocation of proteins between locations within the cells and then slower waves of protein degradation and accumulation. However, for a few proteins, the dynamics of change varied between subpopulations of cells and this related to the phenotypic response to the drug. In cells where the levels of two particular proteins accumulated these cells survived but in other cells the levels of these two proteins fell sharply and these cells then self-destructed [26]. Analysis of gene expression ‘noise’ between individual cells reveals that this can vary by more than three orders of magnitude and that cells can slowly fluctuate between metastable states: when a subpopulation of cells were selected for an extreme expression of a particular protein these cells then slowly repopulated the original distribution of diverse metastable states [27, 28]. If in a population of cancer cells a very small subpopulation exists that expresses high levels of a protein that enabled those cells to survive when treated with a chemotherapy agent, then the tumour could be regenerated after a few rounds of division of these surviving cells.

In response to a stimulus or a change in environment, cell signalling such as calcium fluxes can be initiated within seconds, second messenger signalling and protein interactions and modifications including feedback loops then occur over minutes or hours. Subsequent translocations to the nucleus then result in modulation of multiple gene transcriptions, epigenetic processes and noncoding RNAs with consequent altered translations leading to changes in protein concentrations. Finally, as a consequence of all these changes the result is a cell-fate decision in response to the original stimulus with cell division, death or differentiation but these functional changes may not occur until hours or days after the original stimulus. All of these processes are complex, highly dynamic and noisy. The noise in these signalling and gene expression networks appears to fall into self-organising, stable or metastable patterns analogous to ‘minimum energy equilibrium states’ according to the laws of thermodynamics. The metabolic context may greatly affect the stable states preferred by cancer cells just as energy status is central to thermodynamics. Such stochastic heterogeneity, with its array of stable states, may provide cancer cells some plasticity with a mechanism for decision-making that will determine cell-fate at a tumour level and facilitate a misguided differentiation process enabling cancer cells to acquire a malignant phenotype.

The considerable heterogeneity within tumours presents a formidable challenge and goes a long way to explain many of the difficulties in treating the clinical disease: any treatment may be very effective at targeting most of the tumour cells but if a subclone exists that is resistant to that treatment then however minor the subclone the possibility exists that these cells will repopulate the tumour and the cancer will become resistant to that treatment. This presumably contributes to why the success of so many new targeted molecular therapies is measured in additional months but not years.

## Peto’s paradox

Cell sizes have remained roughly comparable across most metazoans and therefore larger species are comprised of a greater number of cells. As most cancers arise from the normal mutation rate which appears to be comparable across species and as the embryos of all species are initially of similar sizes then it would be expected that the larger the species and the longer its lifespan then the higher the prevalence of cancer. If mutations accumulate because of replication errors at every cell division, then the more cell divisions that occur the greater the risk of acquiring a malignant genotype. Human and mouse embryos are of comparable size but the adult human is around 3,000-fold larger than an adult mouse and lives 40 times longer. If every additional cell division increases the probability of cancer, then humans should have considerably more cancers than mice. Wild mice that are reared within a protected environment however die from cancer at similar rates to humans [29]. Blue whales have a long lifespan and grow to 1,000 times the size of a human but are reported to rarely develop cancer [30]. This would suggest that with the inevitable mutations that would naturally occur with the many more cell divisions needed to grow the extra size of larger animals: the larger animals must have evolved better mechanisms for preventing these from resulting in lethal cancers. Richard Peto [31] originally posed this paradox and although numerous potential mechanisms have been suggested to explain this paradox none have yet been proven [32]. The paradox could be explained by more mutations being required for carcinogenesis in larger animals or larger animals having evolved more tumour suppressor genes; comparisons between mice and humans have however revealed that in both cases the numbers are comparable [33]. One of the most plausible theories for the relatively low rates of cancers in larger species is related to the inverse correlation that exists between body size and metabolic rate [34]. As animals have evolved larger bodies, the physical problems of supplying nutrients and oxygen to more expansive and distant tissues has been compensated for by a lower basal metabolic rate reducing these demands. It has been postulated that as the generation of reactive oxygen species (ROS) is a byproduct of metabolism and ROS can induce DNA damage therefore the reduced metabolic rate in larger animals should result in lower mutation rates [32]. An examination of the frequency of mutations in mouse kidney epithelial cells at the same gene locus that had been examined in humans however revealed that mutation rates are roughly comparable [35]. However, as discussed throughout this review, there is an accumulation of evidence indicating that metabolism is a critical determinant of carcinogenesis and therefore a lower metabolic rate may provide a solution for Peto’s paradox.

## Genes in context

The concept that gene mutations play a determining role in the generation of cancer has become ingrained since the demonstration that the introduction of DNA extracted from cancer cells into normal mouse fibroblasts transformed these cells into cells that could form tumours [36]. It is however well recognised that gene mutations that are known to contribute to cancer within specific tissues may occur exactly the same in other tissues without such an effect and hence the mutations *per se* are not sufficient to cause cancer. The importance of the local tissue environment for a transformed cell to become a cancer is indicated by genetic cancer syndromes caused by highly penetrant mutations; such as Bloom syndrome, neurofibromatosis and BRCA1/2 mutations [37]. Even in such syndromes where there is a clearly identified heritable risk, despite these mutations occurring throughout the whole body, they only result in cancers within specific tissues where presumably the milieu provides the favourable setting. In all other tissues, the presence of these mutations do not result in tumours and therefore by themselves the mutations alone are not sufficient to cause cancer. Even germline mutations in instability genes only result in increased susceptibility to cancers in specific tissues despite being present in every cell and contributing to general DNA maintenance mechanisms. For example mismatch repair (MMR) genes have an identical role in every cell throughout the body but inherited defects in MMR genes result in susceptibility to tumours in the colon and endometrium but not in other tissues with rapidly dividing cells such as the small intestine and bone marrow [38]. Similarly inherited mutations in *BRCA1/2* genes predispose to cancers of the breast, ovary and prostate and to a lesser extent pancreas and colon but not lung or bone [39]. Somatic mutations are also tissue specific in their effects. Mutations in KRAS2 can initiate the development of pancreatic cancer [40] but the same mutations may result in hyperplasia in the colon [41] or borderline lesions that do not progress to carcinomas in the ovary [42].

Stepping back from the genes within a cell to take a broader perspective of the cells themselves, it is also clear that how a cell behaves is dependent on its context within a tissue and this also applies to cells that possess potential cancer contributing mutations. There has been extensive consideration of how a tissue environment affects the behaviour of cancer cells [43]. That the microenvironment is of critical importance is highlighted by different experiments in which cancer cells have been transplanted into normal tissues and the neoplastic phenotype has been shown to be reversible. Exposure of metastatic human melanoma cells to an embryonic zebrafish microenvironment resulted in a re-programming of the melanoma cells to a non-tumourigenic phenotype, indeed melanoma cells implanted into zebrafish embryos were still present 3 months later but remained dormant and unable to form tumours. The same cells transplanted into zebrafish just 2 days later, after morphogenesis and organogenesis were complete, however formed tumours [44]. The injection of the Rous sarcoma virus (RSV) into the wings of chickens also produces large and lethal malignant tumours but injecting RSV into the wings of early chick embryos did not induce tumours and the embryos continued to develop normally; but if those embryonic chicken wings were removed and dissociated in a culture dish then they rapidly developed a transformed phenotype [45]. The effect of a normal tissue environment on cancer cells and how this may change has been elegantly demonstrated by inducing mammary tumours in rats by injection of *N*-nitrosomethylurea (NMU); 50,000 of the resultant cancer cells from these donor rats were then introduced into cleared fat pads of host rats of varying ages and also into rats that had been through two cycles of pregnancy, lactation and involution. Six months after inoculation, tumour incidence in the host rats varied considerably dependent on the age of the host: from 100 % in 52-day-old rats to 18.2 % in 150-day-old rats but no tumours at all occurred in the twice-parous rats [46].

The dogma that mutations alone were sufficient to cause epithelial cancers has also been challenged by experiments in which epithelial and stroma cells were separately exposed to carcinogens. The transplantation of rat mammary epithelial cells that were previously exposed to the NMU into cleared fat pads of rats failed to form tumours but formed phenotypically normal ducts. If the stroma was however exposed to NMU then neoplastic transformation of the mammary epithelial cells was observed regardless of whether the epithelial cells were exposed to NMU or not [47]. When a non-tumourigenic mouse mammary epithelial line was also transplanted into cleared fat pads of mice, tumours were formed in 81 % of mice that had previously been exposed to gamma-radiation prior to the transplantation but only 19 % if the mice had not been irradiated and the tumours were larger and developed earlier in the irradiated hosts [48].

All of these experiments indicate a malignant genotype only results in tumours in certain contexts and that in other contexts the phenotype can be completely reverted despite the same cancer genotype. A malignant genotype itself is therefore not sufficient to generate a tumour but also needs an appropriate context. The same underlying principle was proposed well over a century ago to explain why different tumours predominantly metastasised to very specific tissues. The English surgeon Stephen Paget suggested that the distinct sites of metastasis were not only dependent on the disseminated tumour cells, the ‘seed’, but also depended on the ‘properties of the soil’: the appropriate tissue microenvironment that would enable the seed to grow and develop [49]. From the evidence described above, the same principle appears to apply at the primary site: the cancer genotype will only develop into a tumour phenotype if the tissue microenvironment is appropriate. This same principle has been proposed to explain why occult tumours that normally occur in most people only progress to clinical cancers in relatively few individuals [50]. It may also help explain the long recognised issue of clinical cancer dormancy: following removal of the primary tumour, residual cancer cells can remain in the body for decades without the development of clinical cancer but then relapse after a protracted period of ‘dormancy’ [51]; for example, 20 % of women treated for breast cancer who are disease free, then relapse 7 to 25 years following the original mastectomy with a relatively steady rate of recurrence even after such a long period [52]. This again indicates that cells with a malignant genotype can exist for long periods within the body but only elaborate the malignant phenotype when the context is appropriate. The observation that as rats age the ability of inoculated breast cancer cells to form tumours decreased [46], suggests that the ‘soil’ changes with age. With the inevitable accumulation of genetic damage that naturally occurs with age, such a change could represent a defensive adaptation to prevent cancers from progressing.

## From genes to pathways

Cancer is an acquired genetic disorder due to an accumulation of gene mutations: it has however become clear that the number of mutations that tumours harbour can be very large, as indeed are present in normal tissues, with a very diverse pattern of mutations even in similar tumours [5, 6]. With a recognition of the prevalence of mutations, it has been proposed that even mutations in oncogenes, tumour suppressor genes or genetic instability genes should be regarded just as contributing to cancers rather than as causing cancers [3]. The observation that most cancers do not have an abnormally high mutation rate, but the measured number of mutations is roughly equal to that expected in somatic cells having passed through many rounds of cell division acquiring mutations at the normal mutation rate, indicates that defects in instability genes are not common in the widespread non-hereditary cancers. Germline mutation in these instability genes however result in cancer predisposition and these contribute to the most common syndromes of hereditary breast and colon cancers.

The huge variety of mutations observed in similar tumours originating from the same tissues indicates that many different genotypes can result in the same phenotype (Fig. 3). When this diversity was recognised it was proposed that one explanation could be that the genes were organised into much fewer number of intracellular pathways and it was the disturbance of these pathways that was driving carcinogenesis [53]. When technology was developed such that comprehensive sequencing of genes across the entire genome became feasible; the pattern of mutations observed indeed fitted this model. After analysing the sequences of 20,857 transcripts from 18,191 genes in 11 breast and 11 colorectal cancers the authors characterised the mutational landscape identifying frequently mutated genes that were termed ‘mountains’ and the less frequently mutated genes termed ‘hills’ [6]. The ‘mountains’ were the well-known oncogenes and tumour suppressor genes. These gene mutations occured relatively early in the course of cancers and were not specifically associated with the metastatic stage which is the cause of most cancer deaths [3]. The ‘hills’ were described by the authors as dominating the landscape and the diversity in the ‘hills’ was not hopelessly complex but predominantly grouped into a small number of cell signalling pathways: with the phosphatidylinositol-3 kinase (PI3K) pathway being the most prominent [6]. Genes within the PI3K pathway have been found to be altered in more than 30 % of all colon and breast cancers [54]. From the considerable evidence emanating from studies of the cancer genome it was concluded that it was these ‘signalling pathways rather than individual genes’ that determined the course of tumourigenesis [3]. This indicated a major shift in emphasis in how we should consider the development of these common cancers.

## From pathways to programs

With a large burden of genetic damage naturally accruing as we age, there are many mechanisms that have evolved to prevent these from resulting in fatal cancers and it is now clear that for a cancer to develop there are a number of essential controls that have to be overcome. These were originally described as the six hallmarks of cancer [55], and these have recently been updated with a further four hallmarks [56]. All of the described properties essential for carcinogenesis are not however novel properties specific to cancers but are properties that in some tissues, at some stages of life are perfectly normal; for example, increased cell proliferation, increased energy metabolism, evasion of growth suppressors, evasion of contact-inhibition, evasion of cell death, invasion, metastasis and induction of angiogenesis are all important steps in cancer progression but in early foetal life all of these processes are a normal and essential requirement for development and morphogenesis. Furthermore, during foetal development these processes are all integrated in a developmental program. Indeed one of the early concepts of cancer was that it was initiated from embryonic-like cells or embryonic ‘remnants’ [57] in which this program was reactivated. Some of these processes are maintained in normal tissues throughout the lifespan: for example the constant renewal of epithelial surfaces requires cell division and migration of cells from the basal to the surface layer: indicating that there are varying combinations of properties that may be programmed together. The integrity of the organism is dependent on the ability of epithelial surfaces to repair when wounded. Wound healing involves cell proliferation, migration, inflammation and angiogenesis: all processes fundamental to tumour progression. Indeed tumours have been likened to wounds that do not heal [58] or even to wounds that over-heal [59]. The wounding of an epithelial surface has been reported to result in an instantaneous generation of an electric field that guides cell migration and the wound healing process and this signal is mediated *via* the PI3K pathway [60]. As described in the other sections of this review, this pathway also appears to play an important role in epithelial carcinogenesis.

In amphibians not only can epithelial surfaces be repaired but whole limbs can be regenerated: which indicates that the cells retain the plasticity to be able to reactivate the whole morphogenesis program that normally only operates in early development. This involves the formation of a blastema at the site of injury that then has the ability and plasticity to grow and differentiate into all of the different tissues that can then form a new limb. In species that retain this ability for regeneration, there appears to be an inverse relationship between tissues able to regenerate and those that can support the development of tumours. Studies in newt, salamander and axolot established that it was difficult to induce cancers and these only occurred in tissues that lacked the ability to regenerate and in contrast tissues that could regenerate, such as limbs and tails, were extremely resistant to cancer [61, 62]. Thus, it appears that tissues that retain the plasticity and ability to reactivate the program for morphogenesis do not provide the context, or soil, in which a cancer can progress.

The various hallmarks of cancer are not specific distinguishing properties of cancer cells; they are all active in normal tissues at varying stages throughout the lifespan but in a tightly controlled manner to ensure that tissues throughout the body function in a highly organised manner. What distinguishes cancers is that these programs are reactivated inappropriately and evade the normal controls. For a malignant cell to acquire all of the hallmarks of cancer, a separate defect to overcome all of the many individual controls is not required: it just requires reactivation or co-opting the program that is activated normally during early development or during wound healing.

## From prevalent mutations to prevalent occult cancers

In a similar manner to the general under-appreciation of the prevalence of gene mutations: there is also a lack of appreciation of the true prevalence of cancer in the general population. In reality virtually every individual has small tumours in their body by the time that they get to their sixth or seventh decade of life although in most these lesions never progress to clinical disease. This has been referred to as ‘cancer without disease’ [63]. The prevalence of these latent, occult tumours in the general population has traditionally been assessed by autopsy studies of individuals who had died of causes other than cancer but this evidence has been substantiated for colorectal cancer by population colonoscopy screening studies and for prostate cancer by large cohort studies that have involved the taking of prostate biopsies.

The common cancer with the least available evidence is breast cancer for which only a few, relatively small, autopsy studies have been published [64]. Although the results have been somewhat variable: the largest studies have only been of a couple of hundred women and the methodology has varied considerably in relation to sampling method and the extent to which the breasts were investigated. The prevalence of cancers detected appeared to be strongly dependent on the number of histological sections examined. The most thorough histopathological study of breasts obtained at autopsy in Denmark examined an average of 275 paraffin blocks per breast: for women between the ages of 40 to 50 years not known to suffer from breast cancer when alive: 39 % were found to have ductal carcimonas *in situ* and another 12 % had atypical ductal hyperplasia [65]. Studies examining much smaller numbers of sections found less cancer. In contrast the prevalence of colorectal cancer in the general population has been examined in many autopsy studies and also increasingly in screening colonoscopy studies. A comparison of the two different types of study indicated reasonable agreement with an increase prevalence of adenomas detected with age; by the age of 70 years the mean prevalence of adenomas detected by autopsy studies was 42 % and that detected by colonoscopy studies was 37 % with hyperplastic polyps also detected by autopsy in 28 % and by colonoscopy in 31 % [66]. A recent colonoscopy study investigating subjects at increased risk for colorectal cancer using a magnifying colonoscope found that for individuals in their early 60s, each had a median of 6 aberrant crypt foci, small growths in the colon, and the number was associated with the subsequent risk of colorectal cancer [67].

Even more extensive data are available for occult prostate cancers, again autopsy studies reveal a very high prevalence of latent cancers in the general population [68]. Reports indicate a steady increase with age [69] such that latent cancers were detected in up to 80 % of men aged over 90 years [70]. A recent autopsy study compared American men of African descent with those of European descent. The same pathologists examined prostates obtained from 1,056 men and, despite men of African descent having a 60 % higher incidence of clinical prostate cancer and 2- to 3-fold higher mortality, the rates of latent cancers detected at autopsy were identical irrespective of ancestral descent [71]. Latent cancers were even found in men in their twenties (8 % of men of African descent and 11 % of men of European descent) and by their seventh decade the prevalence had increased to 77 % in men of African descent and 69 % in men of European descent [71]. This study indicates that the higher rates of prostate cancer incidence and mortality in African American men is not due to more cancer initiation. The high incidence in African American men contrasts with the very low incidence within populations of western Africa, from where the ancestors of African American men originate, indicative that the high incidence is likely not due to genetics but more likely due to environmental exposures [72]. Further evidence for the general high prevalence of latent prostate cancers comes from data from biopsies taken from men with normal PSA and digital rectal examinations in the Prostate Cancer Prevention Trial. The prevalence of latent cancers detected from biopsies (15.2 %) was lower than comparable autopsy series (18.5 to 38.8 %) [73, 74], but this difference would be expected because of the under-detection rate when comparing small biopsies with whole-mount prostate gland analysis.

These are examples of studies examining specific tissues but if we consider all epithelial cells throughout the body then it seems clear that all elderly individuals will have one or more latent occult cancers somewhere in their body. Lesions that occur in up to 80 % of all men in the population who survive into old age are, by definition, normal and the evidence clearly indicates that occult tumours within epithelial tissues is a normal feature of aging. This evidence is entirely consistent with the evidence indicating that large numbers of cells will have acquired sufficient mutations to generate a cancer genome in most elderly individuals (Fig. 1).

## Metabolism and the PI3K pathway

**Fig. 4:**
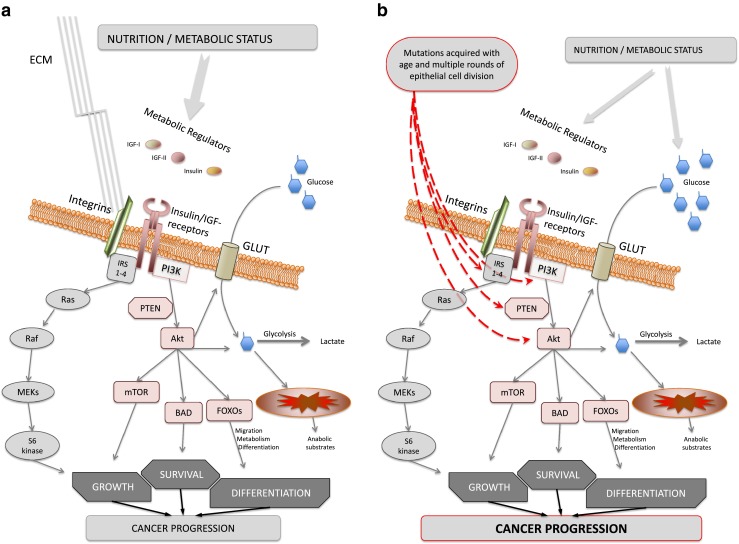
**a** The PI3K pathway is the evolutionary conserved signalling pathway to ensure that growth and development is synchronised to the appropriate metabolic conditions: energy and substrate requiring processes only proceed when a ‘fed state’ is indicated. In metazoans, the insulin/IGF receptor modulates the activity of the PI3K pathway according to social signals indicating metabolic status throughout the organism. Growth and development is regulated, along with nutrient partitioning, and appropriate signals from the extracellular matrix (*ECM*) that are integrated *via* integrin receptors that associate with the insulin/IGF receptors in the cell membrane. **b** Perturbed metabolism and insulin/IGF activity can reprogram cell behaviour *via* modifying FOXO transcription factors: promoting epithelial mesenchymal transition and activating developmental cell programs resulting in cells adopting a metastatic phenotype. Mutations in the PI3K pathway and loss of PTEN can result in constitutive activation of the pathway promoting more autonomous control, less dependent on external signals, and further promotes progression and metastasis

From the above evidence, it is clear that as we age there are many cells in the body that naturally acquire a genotype that could contribute to cancer but the progression of a cancer depends on the disturbance of cell signalling pathways and the reacquisition of a program that enables it to acquire all of the hallmarks of cancer. Programs similar to those active in early development and wound healing need to be activated to overcome the controls that normally maintain tissue homeostasis. Furthermore it seems that the PI3K pathway plays an important role both in wound healing and in tumourigenesis. If we again step back from just considering cancer cells and take a broader view of this pathway and its general role it is clear that it has played a very fundamental role in animals that has been maintained throughout evolution. The ability to control growth and development according to the availability of nutrients provides a survival advantage and therefore has been selectively retained throughout evolution. The ability to delay growth and reproductive development at times when food is scarce enables the organism to survive until more food becomes available and it can then grow, develop and produce offspring when food is again abundant. Evidence has accumulated to indicate that the PI3K pathway provides this control in all species from yeast to mammals. In lower species, such as *Caenorhabditis elegans*, when food is scarce the pathway is switched off and the organism goes into a dormant phase until food is again available and lifespan can be dramatically prolonged to ensure survival through such periods of prolonged famine [75]. The effect on lifespan of suppressing the PI3K pathway appears to have been then conserved throughout evolution [76]. Various forms of the PI3K enzyme exist that are classified into three groups (classes I, II and III). Only one of these forms is present in yeast and is equivalent to mammalian class III PI3K, which in yeast is termed VPS34: this acts as a nutrient sensor and is directly activated by the availability of amino acids and then itself activates mTOR/S6K1 to regulate cell growth and development [77]. In mammals, class 1A PI3K has evolved: this form is not directly activated by nutrients but consists of heterodimers comprising a regulatory subunit and a catalytic subunit that enables the enzyme to be controlled by receptor tyrosine kinases, classically the insulin and insulin-like growth factor receptors (IR and IGF-IR) [78]. This enables systemic signals between the community of cells within metazoans to synchronise these fundamental controls ensuring that all growth and development throughout the organism occurs appropriate to the availability of nutrient substrates. It is not by chance that the insulin/IGF/PI3K pathway plays a fundamental role in regulating both metabolism and growth as it clearly is an advantage to synchronise these two processes and this synchronised control has been maintained throughout evolution. Early human development is also tightly regulated according to nutrient availability by the insulin/IGF axis [79] ensuring that growth and development only proceeds when the cells receive the appropriate signal, activating the PI3K pathway, and this signal provides an indication to the tissues that the organism is in the ‘fed state’. As cancer cells reactivate a program analogous to that active in early development, it is not surprising that this fundamental control is also engaged. Indications that the PI3K pathway is critical for the progression of epithelial cancers suggests that this is indeed the case and that these cancers are more likely to progress if they are receiving a signal that the organism is in the ‘fed state’ and hence energy and substrates are available (Fig. 4a). As the cancer progresses and the tumour cells evade the normal social signals and become more autonomous then this signalling pathway may become constitutively activated in the cell because of genetic or epigenetic alterations (Fig. 4b).

## Cancer metabolism and oncogenes/tumour suppressor genes

A cancer is by definition cells that have acquired a growth advantage: rapid growth is dependent on an increase in supply of energy and of substrates for the biosynthesis of all the macromolecules that are required to build new cells. In normal tissues in an adult, in cells that are not proliferating metabolism is geared up to obtain maximum energy from metabolic fuels. The most available metabolic substrates in the body are glucose and glutamine (which is the most prevalent amino acid). The most efficient generation of energy from glucose occurs by *via* glycolysis to pyruvate which then is fed into the tricarboxylic acid (TCA) cycle in the mitochondria where the carbon is eventually burnt down to carbon dioxide through oxidative phosphorylation. The initial glycolysis yields 2 molecules of ATP from every molecule of glucose and the TCA cycle eventually yields a further 34 molecules of ATP. More than 80 years ago the German biochemist Otto Warburg observed that cancer cells did not metabolise glucose in this way but instead fermented glucose to lactate; a process originally described by Louis Pasteur and thought to occur in the absence of oxygen and therefore no substrate for oxidative phosphorylation. In cancer cells, this however occurs even when adequate oxygen is available and is referred to as aerobic glycolysis. Initially this seemed very inefficient as it yields only 4 molecules of ATP per molecule of glucose. It was however subsequently been realised that this metabolic switch was required in proliferating cells because rather than burning all of the carbon down to CO_2:_ the carbon backbones from glucose were needed to be fed into the TCA cycle where they were required for building all the new macromolecules required to duplicate the cell. In proliferating cells glucose and glutamine must not only supply the energy requirements but must also supply the building blocks and some of these substrates therefore have to be diverted to supply macromolecular precursors such at acetyl-CoA for the synthesis of fatty acids and lipids, glycolytic intermediates for the synthesis of amino acids and ribose for the synthesis of nucleotides. The additional energy required is then obtained by increased flux through glycolysis. This is not only a requirement for proliferating cancer cells, but also for embryonic development and for cells in normal tissues that continue to proliferate throughout adult life. Thus proliferating lymphocytes in response to infection, switch to aerobic glycolysis [80, 81] and during wound healing cells switch to aerobic glycolysis [82]. It was subsequently surmised to be a requirement for enterocytes, lymphocytes, thymocytes, tumour cells and indeed all rapidly dividing cells [83]. The high flux of glucose and glutamine was also suggested to not only provide necessary energy and biosynthetic precursors for cell division but in addition enabled more sensitive regulation of proliferation rather than just relying on regulation of cell metabolism *via* allosteric effects of metabolites on rate-limiting enzymes [83].

Following the description of the ‘Warburg effect’, there was considerable interest over the subsequent decades in this metabolic switch that is fundamental for cancer development. This interest however then waned; as the research focus became dominated by oncogenes and tumour suppressor genes. Interest has however recently been reawakened through findings in a number of different fields [84, 85]. Indeed the importance of the metabolic switch to aerobic glycolysis for proliferation even in normal cells appears to have been rediscovered [86].

The evolutionary advantage, of being responsive to nutrient availability, was originally acquired in single-cell organisms by key enzymes in metabolic pathways that were able to directly sense nutrient concentrations. In mammals, cells within most tissues are however provided with a relatively constant nutrient supply sufficient to support proliferation and the individual cells are no longer autonomous but are part of a large community and their proliferation is accordingly tightly regulated by communal signals. The PI3K pathway appears to be the critical regulator that mediates the response to growth factors and enables the appropriate switch in cell metabolism [78, 87]. The form of PI3K that is present in single cell organisms is directly activated by nutrients whereas the form present in metazoans is activated by growth factor receptors: enabling synchronised response to nutrient availability across the populations of cells within multi-cellular organisms. Activation of the PI3K pathway is sufficient to induce the Warburg effect, switching metabolism to aerobic glycolysis in both normal and cancer cells [88]. Activation of PI3K increases the cell surface abundance of transporters that enhance the uptake of glucose, amino acids and other nutrients; it also activates hexokinase and phosphofructokinase-1 channelling glucose through the glycolytic pathway and in addition it directly stimulates lipogenesis [89] and *via* mTOR it stimulates protein synthesis [90]. The final rate-limiting step of glycolysis, which generates ATP, is catalysed by pyruvate kinase. Pyruvate kinase exists in multiple isoforms: it was proposed that PKM2 was present in embryonic tissues but this was progressively replaced by PKM1 that then became the predominant form in most differentiated cells in adults. However PKM2 may become re-expressed in most cancer cells and counter-intuitively PKM2 has lower enzyme activity than PKM1 [91]. While PKM1 is a constitutively active enzyme, in contrast PKM2 is regulated by tyrosine kinases that induce the release of an allosteric activator (fructose-1,6-bisphosphate) resulting in inhibition of PKM2 activity [92]. The presence of the PKM2 isoform enables the Warburg effect and a switch in metabolism by slowing glycolysis and facilitating the shunting of carbon substrates into biosynthetic pathways [93]. The implication that cancer cell metabolism may be driven by a tumour specific PKM2 raised the prospect of an attractive therapeutic target but then further work indicated that PKM2 was also the predominant isoform present in matched normal tissue [94] as had indeed been observed many years earlier [95].

Following decades when the focus has been dominated by interest in mutations in oncogenes and tumour suppressor genes as drivers of autonomous cancer cell growth, a resurgence of interest in cancer cell metabolism has partly arisen from observations that many of these oncogenes and tumour suppressor genes actually control elements of the metabolic switch that has to occur in cancer cells. The tumour suppressor gene PTEN (phosphatase and tensin homolog deleted on chromosome ten) was originally described as being frequently mutated in brain, breast and prostate cancers [96] and it is also the most frequently deleted gene in many human cancers [78]. It has subsequently become clear that PTEN is a lipid phosphatase that dephosphorylates the product of PI3K and hence directly opposes PI3K. Loss of PTEN therefore results in unopposed PI3K activity and activation of the Warburg effect as described above. Another tumour suppressor gene that is frequently inactivated in human cancers due to mutations is p53 which has been reported to induce TP53-induced glycolysis and apoptosis regulator an enzyme that degrades 2,6-fructose bisphosphate, which is an allosteric activator of phoshofructokinase 1 (PFK1) the enzyme that redirects glucose into the pentose phosphate pathway [97]. Loss of p53 therefore results in increased activity of PFK1 leading in turn to increased glycolysis. At the same time it was shown that p53 also induces cytochrome C oxidase 2 an enzyme that enhances mitochondrial respiration [98]. Therefore loss of p53 results in both reduced mitochondrial respiration and increased glycolysis as per the Warburg effect. In the same year that PTEN was discovered the c-Myc oncogene was also linked to the regulation of tumour metabolism with a report that c-Myc activated lactate dehydrogenase and this was required for tumour growth [99]. It has subsequently become clear that c-Myc regulates many of the enzymes involved in glycolysis [100] and also affects many genes involved in glutamine catabolism [101] and furthermore stimulates mitochondrial biogenesis [102].

Although it has become apparent that many oncogenes and tumour suppressor genes may elicit the Warburg effect in a cell autonomous manner [103], it is clear that the regulation of the metabolic switch enabling cells in metazoans to enter proliferative cycles is fundamentally regulated by growth factor signalling. From the evidence described in the previous sections, it is clear that while gene mutations initiate cancers: for them to then progress, the ‘seeds’ need to be in the right ‘soil’ and a signal indicating that the cells can switch metabolism to that appropriate to a ‘fed state’ is critical for their growth. It seems that many tumours, at least initially, still respond to the communal metabolic signals (particularly the main activators of the PI3K-pathway, insulin and IGFs) and these signals may constitute part of the context that enables the cancer to progress: indicative that the ‘soil’ is fertile. With progression however this pathway often eventually acquires constitutive activation (Fig. 4b).

## Heritability of common epithelial cancers

The extent to which the common cancers are heritable is a further area of general misconception. The genetic contribution to the risk of common diseases has conventionally been assessed in twin-cohort studies comparing the incidence of disease in monozygotic twins (who share all of their genes) with dizygotic twins (who share 50 % of their segregated genes). Such a study of breast, prostate and colorectal cancers in a very large cohort of twins in Scandinavia found the risk attributable to genetic factors to be much lower than that attributable to environmental exposures [104]. The estimated proportion of risk due to heritable factors was 27 % for breast cancer, 42 % for prostate and 35 % for colorectal cancer. The analysis of this study was subsequently updated with genetic susceptibility modelling yielding heritable fractions of 12 to 30 % for breast cancer, 16 to 45 % for prostate cancer and 8 to 27 % for colorectal cancer [105]. The twin studies are based on a number of assumptions and clearly most twins share not just their genes but they also share considerable in respect to common life-exposures. In basic twin models gene-environment interactions are assumed not to exist or they are included as part of the genetic variance inflating heritability estimates [106]. Another approach has been to examine family databases. An analysis of the Swedish Family Cancer Database examined a population of 9.6 million with over 700,000 cancers. The estimated genetic component of risk was 25 % for breast cancer, 13 % for colon cancer and 12 % for cancer of the rectum; prostate cancer was excluded from the analysis [107]. Studies from this large database suggest that at least part of the familial clustering observed for these common cancers could be explained by shared lifestyle-related factors. An elegant alternative approach to estimating the contribution of genetic and environmental influences on cancer was undertaken by assessing cancer risk in individuals who were adopted very early in life. Such individuals have two sets of parents: their biological parents, from whom they inherited all of their genome but shared none of the family environment and their adopted parents, with whom they share no genetic relationship but with whom they shared the family environment. In a cohort of 960 such adoptees the relative risk of cancer was studied in relation to whether one of their parents had died of cancer by the age of 50. The risk of cancer in those individuals where one of their biological parents had died of cancer was 1.19 whereas the relative risk associated with a cancer mortality in one of their adoptive parents was 5.16 [108]. This approach is also complicated by many potential confounding factors but again it suggested that shared exposures had a much greater effect on the risk of these common cancers than shared genes. More than 90 % of the common epithelial cancers are sporadic with only a small proportion being associated with a family history.

## Missing heritability

Following the evidence from classical epidemiology that suggested a significant familial aggregation of the common epithelial cancers and with the advances in sequencing technology there have been many large efforts to identify the genes that may be responsible. Although some genes have been identified such as BRCA1/2 for breast cancer and APC for colorectal cancer that account for a small proportion of these familial cancers the search for additional highly penetrant genes has largely been unsuccessful. After the initial genome-wide association studies investigating associations between single-nucleotide polymorphisms and common diseases, it was acknowledged that only a very small proportion of observed familial clustering was explained [109] and attention began to focus on the ‘missing heritability’ [110]. The major efforts of subsequent work have been to identify undiscovered variants with the hope that rare causative variants could be discovered by increasing the power with next generation sequencing, larger cohorts, consortiums and meta-analyses. These have still however only revealed markers that confer relatively small increments of risk and only explain a small proportion of the observed familial clustering of common diseases. There has also been a realisation that the original expectations were over optimistic, some were based on misplaced preconceptions and others were somewhat naïve: it is clear that the causes of diseases such as the common epithelial cancers are very complex [111]. The contribution of gene–gene, gene–environment and epigenetic variation may all contribute to disease risk and remain challenging to assess. That one gene may act on or modify the effects of another gene (epistasis) has been recognised for a long time but the complexity of gene networks and biochemical systems and how they affect genetic analyses are just beginning to be unravelled [112, 113]. Despite the implications that epistasis may be important, the available data thus far suggest that most gene interactions are merely additive [114] and where multiplicative interactions exist they only contribute a modest effect for prostate cancer [115] and for breast cancer [116].

A further confounding factor has been the heterogeneity of phenotypes with studies examining genotypes with breast, prostate or colorectal cancers although it is now recognised that each of these may represent a broad array of cancers that affect the same tissue and that genes may have different effects on the initiation and progression of these different diseases. Evidence is emerging consistent with both of these issues confounding many of the earlier studies. Low penetrance genetic loci have been found to be associated with specific clinical subtypes of breast cancer consistent with them having different biological origins and hence different heritability [117]. Genetic variants identified to be associated with risk of breast cancer have also been found not to be associated with breast cancer survival indicating that different heritable genes are involved in initiation and progression [118].

The evidence thus far does not indicate that a large component of the missing heritability will be explained by rare variants with large effects or by gene-gene interactions, but there is still much to be learned about the contribution of epigenetic factors. Variations in protein expression and function are not only determined by variations in DNA sequence but may also be affected by many epigenetic mechanisms such as DNA-methylation and histone-modifications, a combination of many small and micro-RNAs, RNA-binding proteins and RNA-editing enzymes. Reports of epigenetic changes in cancer, including all of the above factors, have been increasing exponentially over recent years [119–121]. The IGF-II gene is epigenetically imprinted with only the paternal allele normally expressed but loss of IGF-II imprinting has been found to be a common occurrence in many solid tumours. Furthermore, there have been observations that this loss of imprinting occurs not just in the cancer cells but as a field effect with loss of imprinting also in the surrounding normal tissue. Loss of IGF-II imprinting has been reported in the normal colonic mucosa of around 30 % of patients with colorectal cancer [122] and loss of IGF-II imprinting has been observed also throughout the normal prostate in just under 40 % of men with prostate cancer [123]. The loss of IGF-II imprinting has been associated with augmented activation of the PI3K-pathway [124] and hence the reports of a field epigenetic effect may indicate that this could be a predisposing factor for these cancers. That epigenetic changes may be causally contributing to cancers is supported by the developmental loss of IGF-II imprinting being associated with the most common solid cancer in childhood, Wilms’ tumours [125] and also with the constitutional loss of IGF-II imprinting in Beckwith–Wiedemann syndrome being associated with an increased risk of cancer [126]. Genetic variation may affect a number of these factors; for example single-nucleotide polymorphisms within microRNA have been proposed to affect the risk of breast cancer *via* the effects of these microRNA [127]. There is also considerable evidence that some of the epigenetic modifications may themselves be conserved across generations [128, 129]. The methylation of the IGF-II gene, which is associated with the imprinting of this gene, shows familial clustering across three generations suggesting that genetic factors may play a role in epigenetic modifications of IGF-II [130]. Some of the missing heritability may therefore be due to epigenetic inheritance.

It has however also become evident that at least some forms of epigenetic inheritance are affected by environmental exposures [131, 132]. This additional layer of complexity blurs the distinction between inherited and acquired variation in that environmental exposures may induce epigenetic variations that are expressed several generations later; for example, nutritional exposures during pregnancy can result in transgenerational effects on cancer susceptibility by inducing changes in the DNA-methylating pathways which are passed on down several generations [133]. A high fat diet during pregnancy in mice has been reported to affect phenotype into the third generation of offspring *via* affects on imprinted genes [134]. Supplementing the diets of pregnant mice with methyl donors has also been found to induce DNA hypermethylation which attenuated a predisposition to obesity in successive generations of offspring [135]. This method indicates that some of the heritable component of these cancers may indeed be due to environmental exposures acting across generations *via* epigenetic mechanisms. The interactions between genetic and environmental effects on the epigenome have been elegantly illustrated in studies of monozygotic twins and family cohorts. Identical twins have remarkably similar epigenomes early in life but variations increase with age and twins who spent less of their lives together or who had more differences in natural health and medical history showed the greatest divergence [136, 137]. A study of longitudinal intra-individual changes in DNA-methylation, including a family based cohort, observed both global and gene-specific variations over time but with some familial clustering of methylation change [138]. This could indicate that within families there were common exposures that affected epigenetic changes or that these changes were under genetic control.

In addition to nutritional exposures directly leading to epigenetic changes that could alter the expression of cancer susceptibility genes, it is also clear that nutritional exposures may affect the general systemic metabolic status and hence the hormonal milieu. These hormonal changes may then affect cancer susceptibility or progression by changing the soil within which the cancers develop. It has therefore become clear that much of the heritable component of these common epithelial cancers identified by classical epidemiology approaches may indeed not be because of the directly inherited genes but may actually be because of environmental exposures acting *via* epigenetic pathways that may affect both the seed and the soil across generations of families. Such interactions also complicate the statistical modelling of gene-environment interactions since they violate the assumption of independence: with some of the genetic variance clearly depending on exposures.

## Environmental exposures

In a seminal paper in 1981 Doll and Peto estimated that 30 % of cancer deaths were due to smoking and 35 % were due to diet with a variety of other factors contributing to the remainder [139]. In an update in 1998 Doll reiterated that the incidence of cancer in middle and old age could in principle be reduced by 80–90 % [140]. That many cancers are in principle preventable is implied from the large geographical variations in their incidence. Cancers that are very common in Western societies are relatively very rare in other regions of the world, particularly in Eastern countries [68, 141–144]. These geographical variations cannot be completely explained by differences in ascertainment and it is also clear that they cannot be explained by simple genetic differences since there have been many studies of migrants who have moved between regions of the world were the rates of these cancers are very different. Such studies have consistently observed that cancer rates in migrants soon converge to the cancer rate of the population in their new locale: the time-frame of such changes excludes genetic differences and implicates lifestyle and/or environmental exposures [145].

The incidence of breast cancer in migrants from Italy recently arriving in Australia was half that of native born Australians but in Italians who had been resident for more than 17 years the rate had risen to match that of Australians. Similarly the rates of colorectal cancers in recent arrivals from Italy and Greece were less than half that of Australians but these rates were more than doubled in those migrants resident for more than 17 years [146]. A study of migrants from China, Japan and the Philippines who had settled in California and Hawaii found that breast cancer rates were 30 % higher in those who came from Urban areas in the East compared with rural areas and migrants who had been in the USA for more than 10 years had rates 80 % higher than recent immigrants [147]. The multiethnic cohort study has compared rates of breast, prostate and colon cancer between Japanese and Hawaiian Caucasians and Japanese migrants to Hawaii: the rates of all of these epithelial cancers were considerably lower in the Japanese but increased in first and second generation migrants to Hawaii and indeed for colon cancer had already reached that of Caucasians by the first generation [148]. A review of prostate cancer in Japan and the USA found that incidence rates in Japan and other Asian regions were much lower than that in the USA but rates in migrants to the US from each of these regions were considerably higher than that of their homeland [149]. The incidence of colon, rectal, prostate and breast cancer in China are also considerably lower than that in the USA but the incidence in Chinese-Americans approach that of Americans [143]. The diets traditionally consumed in China and Japan were very different from that in the USA: in the mid-1950s, the per capita consumption of red meat in the USA was 254 g/day compared with 28 g/day in China and 14 g/day in Japan; also, milk consumed in the USA was 530–670 g/day compared with only 6 g/day in China and 25 g/day in Japan. By contrast, the majority of calories in the diets in China and Japan were from cereals and grains with more than double the intake compared with the USA [143, 144]. Although restricted calorie intake is associated with lower cancer (as discussed below), the geographical differences are more complicated than simple differences in calorie intake. A comprehensive study of diet and lifestyle in 6,500 adults in 130 villages across rural China found that the calorie intake for the average Chinese man was about 30 % higher when compared with an average American man of the same weight [150]. The Chinese men were however much leaner with a diet low in animal fat and protein but high in fibre and rich in plant-based foods. The low BMI in the Chinese men (around 25 % lower than American men) was mainly attributable to high energy-expenditure and much greater physical activity. The geographical lifestyle differences therefore probably relate to overall differences in energy balance and composition of diet rather than to simple calorie intake differences.

Similar implications can be learned from countries where lifestyles have been rapidly changing: Japan was virtually closed to Western influences until the 1950s since when there has been a remarkable Westernisation of dietary habits [151]. This change in exposure has been accompanied by a rapid increase in the height of Japanese girls followed, with a 30-year lag period, by a parallel increase in incidence of breast cancer [152]. This implies that changing environmental exposures that resulted in increased childhood growth may also have influenced the subsequent risk of this cancer. A similar rise in the incidence of prostate cancer throughout Asian countries has been observed and associated with the gradual Westernisation of those countries [153]. If the effect of environmental exposures was on cancer initiation, then the rates of latent cancers would be expected to increase in migrants who move from regions of the world with low incidence of cancers to regions with high incidence. The available data however indicate that the prevalence of latent prostate cancers were similar in Japanese migrants to Hawaii compared with indigenous Japanese [154] despite the increase in clinical cancers. The large changes throughout society in Japan have been accompanied by a modest increase in the prevalence of latent prostate cancers; the age adjusted rate was estimated as 22.5 % from an autopsy series between 1965 and 1979 and had increased to 34.6 % from a similar series between 1982 and 1986 [155]. This was then comparable to the rate of 34.6 % in white men in the USA [156] despite the incidence of clinical prostate cancers being vastly different at around 10-fold higher in the USA than in Japan [149]. Together these observations imply that environmental exposures may have a relatively small effect on the initiation of latent cancers but a large effect on the progression of cancers to clinical disease.

**Fig. 5:**
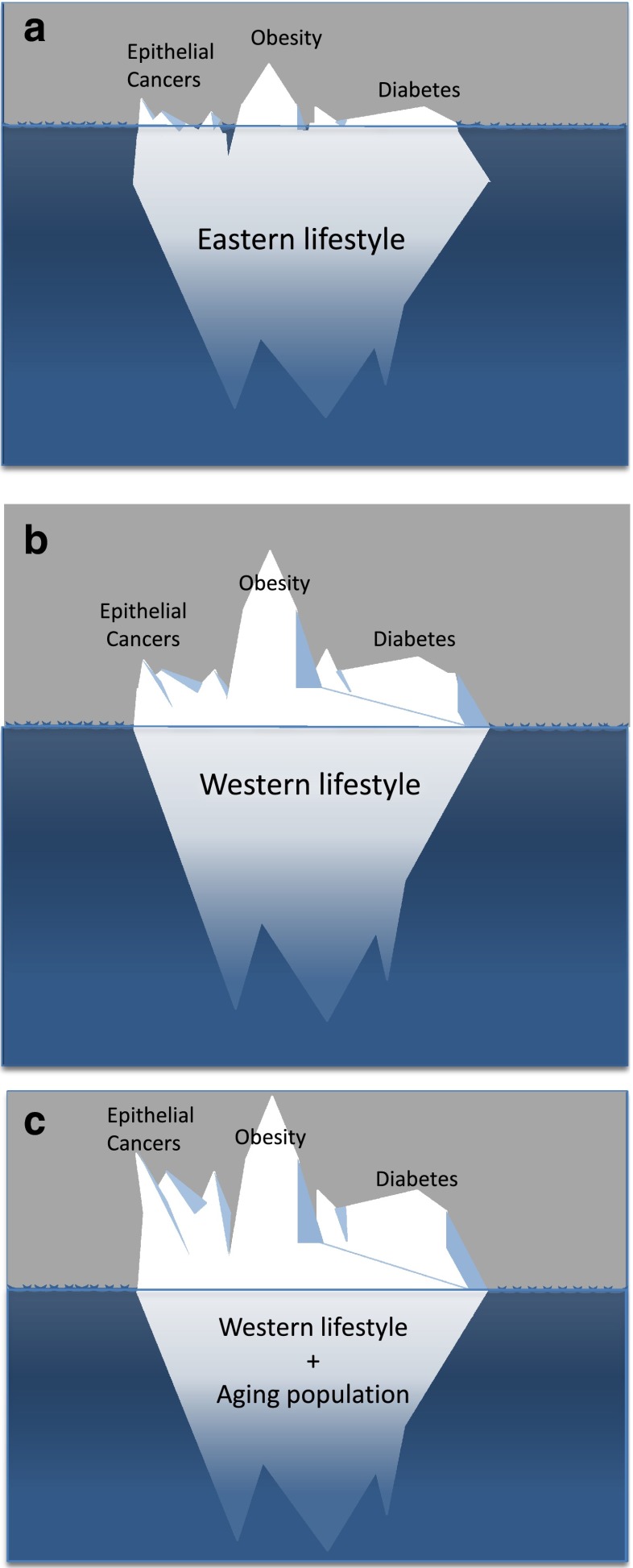
**a** A pre-industrialisation lifestyle, that is still prevalent in some Eastern societies, is associated with limited nutritional intake and high-energy expenditure. Metabolism is then maintained in balance as apparent by the low rates of metabolic disorders, such as obesity and diabetes, in such societies. With aging, oncogenic mutations naturally accrue in rapidly dividing epithelial tissues and as a consequence occult cancers appear; but with metabolism in balance the ‘soil’ is not fertile and the ‘seeds’ are not stimulated by endocrine signals and do not progress to clinical disease. The latent oncogenic potential still accumulates with aging but like the bulk of an iceberg it remains submerged. **b** The adoption of a Westernised lifestyle with excess nutritional intake and a sedentary lifestyle results in energy-imbalance perturbing metabolic control as reflected by the increasing prevalence of obesity and diabetes. This alters the internal ecosystem within the body such that the naturally occurring occult cancers are given signals to progress, that together with a more fertile ‘soil’, results in more of them developing to clinical cancers. **c** When the adoption of a Western lifestyle is combined with the control of life-shortening conditions then there is a shift in demographics with more of the population living longer into old age. This has exacerbated the prevalence of clinical cancers as the occult cancers, exposed to perturbed metabolic signals, have even longer to progress to clinical disease. The large burden of oncogenic potential that naturally accumulates with age then emerges as an epidemic of epithelial cancers: as the tip of this ‘iceberg’ becomes even more exposed

With exposures that are still prevalent in some Eastern societies the epithelial cancers are relatively very rare, as are obesity and diabetes: indicating that metabolic homeostasis is maintained (Fig. 5a). In those individuals surviving into old age, such as in Japan, the initiating mutations still accrue and the occult neoplasias occur but these do not progess to clinical disease. With the metabolic pertubations associated with a Western lifestyle however these progress and become clinically evident (Fig. 5b). In developed societies, the control of previously life-shortening conditions has also shifted demographics with many more individuals surviving into old age. In the rapidly expanding older generations there is then longer time and a more fertile soil for the prevalent occult neoplasias to progress to clinical cancers and the tip of the iceberg becomes even more exposed (Fig. 5c).

With the identification of highly penetrant genes that conferred an extremely high risk of specific cancers, it was generally considered that in such cases the contribution of genetic susceptibility would make environmental influences of little importance. Evidence has emerged however indicating that environmental exposures may still have a dominant role even when there is a clearly identified strong heritable risk, such as with BRCA1/2 mutations that are thought to promote genomic instability. Within a community of over 1,000 Ashkenazi Jewish women with inherited mutations in BRCA1/2, the risk of developing clinical breast cancer by the age of 50 was 24 % in those born before 1940 but increased to 67 % in those born after 1940, with physical exercise and obesity being factors determining cancer onset [157]. Similarly for women in Iceland with BRCA2 mutations the risk of developing breast cancer by the age of 70 was 18.6 % in 1920 and had risen to 71.9 % by 2002; over the same time period in the general population the increased risk was from 1.8 to 7.5 % [158]. The relative temporal increase in cancer risk because of environmental changes was therefore approximately the same in women with a clearly identified heritable risk as it was for women with sporadic cancers. The risk associated with BRCA mutations is so high that women are now recommended prophylactic total mastectomy, but a century ago the risk in women with the same mutations would have been approximately equivalent to that of women in the general population of North America and Western Europe today. Recent studies of French-Canadian families with inherited BRCA1/2 mutations have examined the aspects of lifestyle associated with breast cancer risk and found that total energy intake and weight gain since the age of 18 were positively associated with risk [159] and dietary vegetable and fruit diversity were negatively associated [160]. These studies indicate that exposures that determine progression to clinical disease can have a large effect and operate independently of the initiating factors.

As the evidence indicates that exposures affect the progression of common epithelial cancers, an important secondary question is when such exposures are having this effect. This is important if interventions are to be designed to impact on the course of these diseases. The question is confounded by the believed, but uncertain, long time frame between initiation and the clinical presentation of these cancers that may occur decades later. Direct information is scarce but there is evidence related to a number of surrogate markers of exposure. There has been considerable interest in exposures during critical developmental periods early in life in relation to other chronic disorders in adults, principally metabolic and cardiovascular disorders. The Developmental Origins of Adult Health and Disease hypothesis proposes that foetal nutrition and other exposures result in adaptations of placental and foetal hormones and that when this occurred during critical periods during gestation this could result in the reprogramming of key metabolic hormones such as insulin, IGFs and growth hormone with long-term consequences on metabolic and cardiovascular health in later adult life [161, 162]. That the risk of cancer may be affected by early exposures is suggested by the associations observed with proxys of early exposures such as size at birth, childhood growth and height. Birthweight or size, which are proxys for *in utero* growth and prenatal exposures, have been found to be positively associated with the subsequent risk of breast [163], prostate [164] and colorectal [165] cancers. Adult height, which is determined by childhood growth and the tempo of sexual development and is a proxy for childhood exposures has also been positively associated with these three common epithelial cancers [166]. Studies of breast cancer have established that both birthweight and childhood growth have positive associations with risk [167] with probable independent effects [168]. The largest study of a cohort of 117,415 women found that the pubertal growth spurt had a particularly strong effect and after adjusting for age at peak growth, the age at menarche was no longer related, despite the wide belief that this is a strong determinant of breast cancer risk [169]. These studies could indicate that early exposures are affecting the tissues or cells predisposing to subsequent cancer development or they could indicate that the early exposures are affecting the soil in which the subsequent cancers develop.

There is accumulating evidence that nutritional exposures in early life result in the resetting of hormonal systems with a programming effect on hormonal levels throughout the rest of life. A long-term follow-up of a randomised trial of milk supplementation given during pregnancy and throughout infancy up to the age of 5 years found that serum IGF-I levels measured in the subjects 20 years after the end of the supplementation were reduced compared with subjects not receiving the additional milk [170]. Milk consumption during childhood is positively associated with IGF-I levels [171] and therefore the long term programming effect is opposite to the acute effect. Evidence of an even more long-term effect was obtained in a follow-up of a cohort studied in the 1930s, subjects from families with high milk consumption in the 1930s (who were then children with an average age 6.5 years) had lower circulating IGF-I levels when measured in blood samples taken 65 years later [172]. Further evidence linking nutrition in early life with IGF-I levels measured much later in adulthood was obtained in a long-term follow-up of people exposed to the 1944–1945 Dutch Famine. This again provided evidence for a paradoxical enduring effect opposite to the well known short-term effect of reduced nutrition with girls who were seriously deprived of food for almost 6 months at a median age of 12 years having higher levels of serum IGF-I when measured 51 years later [173]. In a follow-up study examining breast cancer in women exposed to the Dutch Famine, a 48 % increased risk was found in women severely exposed and a 13 % increase in women moderately exposed: the greatest risk (101 % increased) was observed in the women who were severely exposed between the ages of 2 to 9 years [174]. Together, these studies indicated that nutritional deprivation in childhood resulted in reprogramming of systemic hormones with higher adult levels of IGF-I and also increased the risk of breast cancer decades later.

It appears that for most of the common epithelial cancers, decades may elapse between initiation and clinical presentation [4] and it is likely that the effect of perturbed metabolism, consequent to a Western lifestyle, is manifest throughout but with an amplifying impact in the latter more accelerated stages (Fig. 2). This may be analogous to cardiovascular disease where the initiation of atherosclerotic plaques can be detected in autopsies of subjects in their 20s but the clinical consequences of lifestyle exposures only become evident decades later.

## Evidence from intermediate biomarkers of metabolic exposure

Further evidence that metabolic status may influence cancer has come from population studies examining associations between circulating measures of metabolic biomarkers and the risk of subsequently developing cancers. The circulating levels of IGF-I have proven particularly attractive for such studies because these levels are determined by nutritional and metabolic status and hence could represent a modifiable risk factor. The circulating levels are also relatively stable throughout the day and can therefore be measured in untimed blood samples that may be available in many epidemiology studies. In addition, the levels of IGF-I within individuals seem to track over a number of years and therefore reflect long-term exposures. This enables prospective epidemiology where inter-individual variation in IGF-I levels can be used as a measure of exposure and related to subsequent outcomes, such as the risk of cancer or the risk of progression. There are also measures of serum insulin but as circulating insulin levels fluctuate rapidly throughout the day inter-individual variations are best examined in timed, fasting blood samples which are not so readily available from population studies; to overcome this some studies have measured serum levels of C-peptide as a more chronic measure of insulin secretion. For IGF-I, although there has been some heterogeneity with findings, there have been a considerable number of large studies with reasonably consistent findings. There are many reasons why there have been heterogeneity of findings: there are a number of analytical pitfalls with measuring IGF concentrations that can lead to problems for those inexperienced with these assays. There has also been considerable heterogeneity in study design with some prospective studies examining associations between IGF-I and subsequent cancer risk and others being cohort studies where associations may reflect the host response to cancer. Theoretically, associations found in cohort studies could be confounded if serum IGF-I levels were affected by IGF-I being produced in the tumour itself, but this would be extremely unlikely due to the known dynamics of the systemic IGF-system and the evidence in humans confirms that this is generally not the case. There are also issues of phenotype heterogeneity with studies examining cohorts suffering from prostate or breast cancer but individuals within these cohorts having very different forms of cancer.

Despite their attractions, the interpretation of circulating IGF concentrations is far from straightforward as the relationship between serum concentration and tissue production or tissue bioactivity is far from simple. There are no intracellular stores of IGF-I in the body and the very high serum concentration appears to primarily represent a circulating store, maintained by high affinity binding proteins that considerably slow clearance. There is then a complex interplay between various factors that determines the availability of IGF-I from this store and its activity in tissues with local tissue production confounding interpretation further [175]. Despite all of these limitations, robust associations have been observed between circulating IGF-I concentration and the three common epithelial cancers. For breast cancer, positive associations between circulating IGF-I and risk of cancer have been reported in meta-analyses, systematic reviews and in a large pooled analysis of 17 prospective studies [176, 177]. For prostate cancer similar associations have also been found in meta-analyses, systematic reviews and a pooled analysis of 12 prospective studies [178, 179]. A number of studies have also reported positive associations between circulating IGF-I levels and the risk of colorectal cancer and these findings also appear to be relatively robust as indicated in a recent meta-analysis [180].

There is also evidence from population studies of prostate cancer that suggest that the association with circulating IGF-I is not because of an effect on cancer initiation but reflects an effect on the risk of progression to clinically relevant disease. In a meta-analysis that stratified all published studies according to those conducted within PSA-screened populations (the vast majority of cancers that are PSA-screen detected are occult latent cancers) and those studies that involved routine clinically presenting cases: the association with IGF-I remained for the clinically detected cancers but no association with IGF-I was observed in the PSA-screened populations [181]. A large prospective study of nearly 3,000 PSA-detected prostate cancers also found no evidence of an association between risk of cancer and IGF-I [181]. However, in 909 of these cases who were then assigned to active monitoring, there was evidence of an association with biochemical progression as assessed by PSA doubling times after a mean of 4 years follow-up [182]. In a study of 396 men with advanced prostate cancers, there was an even stronger association between IGF-I and prostate cancer-specific mortality [183]. If IGF-I is a marker of nutritional/metabolic status then the lack of association of IGF-I with PSA-detected cancers, the majority of which are indolent, is consistent with the autopsy studies that indicate that lifestyle exposures have relatively little effect on the prevalence of occult cancers. The associations of IGF-I with progression and mortality however are consistent with the population studies that indicate that such exposures have a large effect on the progression to clinical disease.

Reasonably consistent associations have also been found for more direct measures of metabolic status such as glucose and insulin levels. There have been some reports of moderate associations between circulating levels of C-peptide and the incidence of breast cancer [184, 185]. Although many studies have not found such an association but a stronger association has been reported with breast cancer mortality [186] that would be consistent with an effect on disease progression. For prostate cancer, associations have been reported between C-peptide and the risk of high-grade cancers [187] and prostate cancer mortality [188] again supporting a role in cancer progression. With colorectal cancer there have been several reports that higher C-peptide confer a greater risk of adenoma [189–191] implying a possible role in early adenoma formation. Associations between IGF-I and the appearance of adenomatous polyps [192] and between IGF-I and fasting serum insulin levels and the presence and advancement of adenomatous polyps [193] again support a role for metabolism in the early development and advancement of these precancerous lesions.

Although the many studies examining these individual markers provide accumulating evidence: an even stronger picture is obtained from a holistic approach considering all of the evidence together; for example for colorectal cancer there is evidence from cell biology; animal models; clinical evidence such as the association between acromegaly (which is characterised by high IGF-I levels) and colorectal cancer [194]; the blood biomarker associations described above; epidemiology relating height, body mass index and physical activity; associations with metabolic disorders such as diabetes and obesity and a factorial analysis of dietary intake associating a Western diet with colon cancer [195]. When all of this evidence is considered together, the story becomes even more compelling linking lifestyle, metabolism and the risk of these epithelial cancers [196]. This raises the feasibility that a better understanding could lead to lifestyle modifications that would render these cancers rare in populations just as they were in societies before industrialisation and Westernisation.

## Co-morbidity associations

The major change in energy balance consequent to the adoption of a Western lifestyle has resulted in epidemics of metabolic disorders especially obesity and diabetes. It has also been recognised for many years that the geographical variations in the common epithelial cancers closely parallels the spread of these metabolic disorders. The worldwide tally for those obese is approaching 500 million and rising rapidly. There is a similar rise in type 2 diabetes: increasing 4-fold over the last 30 years in the USA. With such large numbers affected any associated increase in risk of cancers represents a major public health concern. A prospective study of 900,000 Americans reported a decade ago that increased body-mass index was significantly associated with higher rates of death from colorectal, breast, prostate and a number of other cancers [197]. Since then there have been many other reports of population studies, meta-analyses and systematic reviews reporting similar associations with obesity and recently also increasingly with diabetes. There are many complications confounding the interpretation of such associations in addition to the issues discussed earlier of cancer heterogeneity and distinguishing between associations with risk of initiation or disease progression. Obesity itself has heterogeneous features that may influence cancers confounding the effects of the underlying metabolic disturbance; for example, steroid metabolism is altered, in particular because of the high expression of aromatase in adipocytes converting androgens to estrogens. This may contribute little to estrogen exposure to tissues in premenopausal women but contribute significantly in postmenopausal women and may clearly have different effects on prostate cancer in men compared with breast cancer in women. Associations with type 2 diabetes may also be confounded by many factors associated with that condition. Early disease is characterised by insulin resistance, and epithelial tissues may be exposed to raised insulin levels but with more advanced diabetes the pancreas may fail resulting in low insulin exposure but if systemic insulin therapy is administered then exposure may again be increased. In population studies different individuals may be on different treatment regimens that may themselves affect cancer development and progression in different ways. One of the most common drugs used for diabetes is metformin that, as discussed in the next section, accumulating evidence indicates may be protective against epithelial cancers whereas other therapeutic options such as insulin analogues may not have such an effect. Many of the early population studies did not discriminate between stage of diabetes or treatment regimens. In the light of all of these potential confounding factors, the relatively consistent associations reported from population studies are probably underestimating the true effect of perturbed metabolism itself. It is also emerging that associations with cancer progression are even more robust than those with incident disease risk. Prognosis and mortality are worse for patients with diabetes for both colorectal cancer [198] and breast cancer [199]. In women with breast cancer, a high level of blood glucose was associated with poor response to chemotherapy [200], consistent with *in vitro* work demonstrating that hyperglycemia conferred resistance of breast cancer cells to chemotherapy [201]. In potential contradiction, diabetes has consistently been reported to be associated with a reduced risk of the incidence of prostate cancer [202] although the association appears to be strongest in studies in the USA [202]. There are a number of potential confounding issues that may help explain this anomaly, particularly relating to detection and population screening as several studies have reported that PSA levels are lower in subjects with diabetes [203]. In contrast to the effect on incidence however there are reasonably consistent reports that pre-existing diabetes is associated with a poorer prognosis for men with prostate cancer [204] and for those with diabetes poorer glycemic control is associated with aggressive cancer [205]. Considering the high prevalence of these metabolic disorders in the general population, an increasing number of patients presenting with cancer have one of these co-morbidities and this has important implications for prognosis, treatment response and survivorship.

## Progression and metastasis

The accumulating evidence that metabolic disorders may have a greater effect on prognosis and survival than on cancer incidence implies that metabolic status may impact on the processes of progression and metastasis. These inferences have recently been supported by observations that men who have been treated for prostate cancer who have elevated blood glucose levels are at 50 % greater risk of disease recurrence compared with men with normal glucose [206]. Hyperglycemia in women with advanced breast cancer was found to be associated also with poorer outcome and increased mortality [207]. As metastases are responsible for around 90 % of all cancer-related deaths [208], the identification of modifiable factors that contribute to metastasis could be extremely important.

The dissemination of tumour cells from the primary site to establish metastatic lesions at other locations around the body is a complex multistage process; the understanding of which has seen major advances over recent years but with some critical questions remaining unanswered [209, 210]. For a cancer to metastasise, tumour cells need to detach from the primary site: this involves acquiring increased motility and an ability to break-down extracellular matrix (ECM). The detached cells need to enter the circulation *via* intravasation into existing or newly formed blood or lymph vessels. The cells need to survive in the circulation and then extravasate from the circulation at a secondary site. Here, the cells encounter a microenvironment that at least initially will be very different from that in the tumour from which they departed and the cells need to survive in this new environment. The cells may just survive here in a relatively dormant state for prolonged periods that may last many years but eventually must resume growth at this secondary site to form a metastasis or secondary tumour. A number of processes have been suggested to contribute to these events. A change in the differentiation status of the epithelial cell referred to as epithelial mesenchymal transition (EMT) has been heavily implicated [211]. This involves loss of cell polarity and the cell-cell adhesion properties characteristic of epithelial cells and acquisition of increased motility, an ability to invade through ECM and resistance to the apoptosis normally induced by detachment (anoikis), properties associated with mesenchymal cells. It has been suggested that EMT may play a pivotal role in driving carcinomas to a more malignant stage [212]. The formation of new blood vessels *via* angiogenesis has been proposed as not only important for the supply of oxygen and nutrients to the tumour but also for providing the tumour cells access to the circulation. The most enduring concept regarding metastasis has been the ‘seed and soil’ hypothesis originally proposed more than a century ago [49]: this proposes that metastases only develop in specific sites where the right context is provided with the appropriate microenvironment. The concept of an appropriate soil to support a potential metastatic lesion has subsequently evolved with an accumulation of evidence supporting the existence and development of metastatic niches that provide such an appropriate microenvironment for the survival and outgrowth of disseminated tumour cells [213]. Indeed recent evidence has suggested that the soil may be prepared in advance with the creation of a premetastatic niche before the first tumour cells actually arrive. Using experimental models, it has been shown that a distant primary tumour can induce the mobilisation of bone marrow derived progenitor cells that cluster in tissues and change the local environment creating a suitable niche [214]. A distant tumour in mice has been reported to induce upregulation of inflammatory chemokines and the recruitment of myeloid cells to promote the formation of a prematastatic niche [215].

There are clearly many barriers that have to be overcome for a tumour to spread throughout the body. Individual adaptations or acquired mutations may not however be required to overcome each of these barriers. Many normal cells behave in a similar manner during early development and even into adulthood with complex patterns of migration around the body and invasion into tissues. In early development, cells migrate from the surface of an embryo to form the mesoderm during gastrulation, neural crest cells undergo EMT and invade through tissues as the progenitor cells that develop into neurons and glia in the peripheral nervous system and other cells such as melanocytes: similar behaviour is also seen in organogenesis [212, 216]. In adults, bone marrow-derived progenitor cells migrate to peripheral tissues to form myeloid, endothelial and mesenchymal cells. Many of the same features, including angiogenesis, are also activated during wound healing. Similar programs of cell behaviour may be inappropriately reactivated in cancer cells enabling them to acquire a metastatic phenotype [211].

It is clear that the genetic factors that initiate oncogenic transformation are in themselves generally not sufficient for the development of metastases as evidenced by the observations that most mouse subcutaneous xenograft models and many oncogene-driven mouse models fail to form metastatic lesions [210, 217]. The implications from the clinical reports that metabolic disorders may adversely affect progression and survival have been supported by various experimental models. In a model of xenografts formed from Lewis lung cancer cells; when the mice were fed with either a high fat or a high sugar diet, either of these diets both more than doubled the rate of metastases [218]. In a orthotopic syngeneic mouse model of triple negative breast cancer, a high energy diet promoted tumour growth, induced lung priming by bone marrow-derived myeloid cells and considerably increased the number of lung metastases [219]. In an autochthonous mouse model of prostate cancer, a Western-type diet was also shown to increase the number of lung metastases [220]. Several mechanisms have been proposed to explain the effects of diet and metabolic status on tumour progression and metastasis. Adipokines released from fat cells are a potential mediator although studies in fatless mice that have undetectable levels of adipokines have shown that diabetes still enhances tumour formation although effects on metastasis have not yet been reported [221]. From the discussions above, it is clear that the insulin/IGF system are prime suspects because of their conserved function in integrating tissue metabolism and growth. In a series of studies in which two different strategies were used to induce mammary tumours in mice in which different genetic manipulations were used to render the mice insulin resistant: it was shown that the insulin resistance consistently increased the number of lung metastases [222–224]. In a mouse model with transplanted colorectal tumours, systemic hyperglycemia was found to increase circulating levels of IGF-I and tumour expression of vascular endothelial growth factor (VEGF) a critical mediator of angiogenesis [225]. In most cancers that have been examined IGF-I has been found to be a potent regulator of VEGF expression [226]. Inactivation of hepatic expression of IGF-I, resulting in a 75 % reduction in its circulating concentration, significantly reduced metastasis to the liver in an orthotopic model of colon cancer [227]. In a further study, diet-induced obesity was found to result in an inflammatory response in the liver in control normal mice and this was associated with an increased incidence of hepatic metastasis following direct intrasplenic/portal inoculation of colon carcinoma cells [228]. The effects of obesity on this inflammatory response in the liver and on the enhanced efficiency of metastasis were not however seen in mice in which hepatic IGF-I expression was negated [228]. This implied that IGF-I was critical for sustaining an obesity-induced metastatic niche in the liver. That humoral factors mediate the effects of obesity is supported by observations that when prostate cancer cells were exposed to serum taken from mice with diet-induced obesity an increase in cell migration, invasion and markers of EMT were observed relative to cells exposed to serum from control mice [229]. An inflammatory microenvironment may recapitulate the tumour-stroma interactions in the primary tumour and support the development of a metastatic niche and in addition it may modulate EMT [213]. A number of inflammatory mediators and cytokines may facilitate metastases by promoting angiogenesis, cell migration and invasion and inhibiting apoptosis [230]. This experimental evidence together with the clinical observations indicates that metastases may be critically affected by the metabolic context within the body.

These studies confirm that perturbed metabolism is associated with enhanced cancer progression and metastasis: other recent findings indicate that altered cell metabolism may indeed initiate these events. Induction of the Warburg metabolic switch in human cancer cells, by genetic silencing of the respiratory enzyme citrate synthase, resulted in greatly increased glycolytic metabolism and this was reported to induce EMT and enhance metastases in an mouse xenograft model [231]. Reversal of the Warburg effect by silencing of expression of pyruvate dehydrogenase kinase 1 in breast cancer cells also restored their sensitivity to anoikis when detached and reduced lung metastasis by 5-fold when they were inoculated in a mouse model [232]. It has long been recognised that developmental EMT can be induced by exposure to hyperglycemia, and it has recently been demonstrated that exposure to hyperglycemia can similarly induce EMT of human lung adenocarcinoma cells [233]. Accumulating clinical studies indicate that the experimental evidence implicating EMT in cancer dissemination and metastasis may also apply to human cancers [211]. This has recently been further supported by a study that showed that circulating breast tumour cells were enriched in cells expressing mesenchymal markers whereas mesenchymal markers were only rarely found in cells from the matched primary tumours [234].

Taken together, these studies indicate that exposure to adverse metabolic conditions can induce EMT and initiate the whole program necessary for metastatic progression. Metastases could be facilitated by processes such as EMT that drive cancer cells into behaviour patterns that are similar to that seen in normal cells during early development and organogenesis. Early development is critically dependent on adequate supply of nutrients with the conserved insulin/IGF signalling pathway regulating cell growth, survival and metabolism in an integrated manner by switching on and off a program of appropriate genes *via* a family of gene regulators, the forkhead box O (FOXO) transcription factors [235] (Fig. 4). There are at least 43 distinct members of the FOXO family of proteins and several of these have been implicated in the regulation of EMT [211]. Recent evidence indicates that breast cancer prognosis is associated with a differential pattern of estrogen receptor (ER) nuclear binding to genes that was determined by a reprogramming of ER binding to these genes by FOXA1 (a FOXO transcription factor); ER and FOXA1 were also found to be co-expressed in samples from metastatic lesions obtained from women with advanced disease [236]. FOXA1 plays a crucial role in developmental ductal morphogenesis in the breast and it has recently been demonstrated that it is a critical mediator of IGF-I effects on breast cancer cell growth and survival [237]. In addition to enabling gene expression, FOXA1 can also repress genes by competing with another FOXO protein, FOXA2, and the pattern of genes activated or repressed is then determined by the relative levels of FOXA1/FOXA2 and this ratio is modulated by insulin/IGF signalling [238]. FOXA2 inhibits EMT [239] and hence FOXA1 by competing with FOXA2 could facilitate EMT. FOXA2 has also been identified as a critical regulator of colorectal cancer metastasis to the liver [240]. Another related transcription factor FOXC1 was associated with the EMT observed in disseminated circulating breast cancer cells [234] and has been linked to the induction of EMT in breast cancer cells [241, 242]. FOXC1 has also been reported to promote the invasiveness of breast cancer cells by inducing the expression of matrix metalloprotease 7 an enzyme known to be able to degrade components of the ECM [243].

A key event in EMT is the loss of E-cadherin which connects neighbouring epithelial cells and hence its loss enables cell migration and invasion [244]. E-cadherin also however acts as a negative regulator of the WNT signalling cascade by controlling the cellular localisation of the critical mediator β-catenin. β-catenin associates in a complex with E-cadherin at the cell membrane and the loss of E-cadherin may permit the translocation of β-catenin to the nucleus where it can induce the expression of genes that promote an invasive phenotype. There is considerable cross-talk between WNT and IGF signalling: IGF-I is induced by, and in turn activates, WNT signalling in a feed-forward loop [244]. This cross-talk with metabolic regulators may enable the acceleration of EMT in compromised metabolic conditions and hence promote metastasis.

Measures of dissemination of tumour cells into the circulation indicate that more than a million cells can be shed daily per gram of tumour [245]. Experimental models however also indicate that the efficiency of metastasis is very low, generally estimated to be less than 0.01 % [4]. The ‘seeds’ appear to be extremely plentiful and the rate limiting factors are the survival of the seeds in the circulation, their ability to extravasate into secondary sites and the suitability of the soil at these sites. With such a low efficiency process, any factor that has even a small effect could have a very important impact on metastasis. The different strands of emerging evidence indicate that metabolic exposures and metabolic regulators may promote these metastatic processes and hence could be important determinants of the progression of epithelial cancers, and consequently mortality.

An alternative hypothesis for how environmental exposures associated with a Western lifestyle could contribute to the prevalence and progression of epithelial cancers stems from the ‘hygiene hypothesis’. A traditional lifestyle, as experienced throughout most of human evolution, was characterised by continuous exposure to environmental microbiota that induced and strengthened the adaptive immune system. This could help prevent the establishment of chronic tissue inflammation that, as described above, may contribute to the creation of metastatic niches. A Western lifestyle, associated with urban living in a microbe-poor environment may however compromise the development of the adaptive immune system and hence promote the creation of metastatic niches [230]. The effects of lack of exposure to environmental microbes and exposure to a lifestyle that compromises metabolic controls are not mutually exclusive. Indeed the considerable cross-talk between metabolic and inflammatory mediators, as mentioned above, could result in these exposures having a compounded effect on cancer progression.

## Metabolic interventions

Since the realisation that diet may contribute to a large proportion of common cancers, there have been many intervention studies investigating the role of various dietary factors that may impact on cancer risk. Although there is convincing evidence linking some dietary components with specific cancers, there have not been major breakthroughs to explain the majority of the effects of nutrition on cancer rates. This is undoubtedly at least partly due to the complexities of diet and the inaccuracies of techniques available to measure nutrition. Comparisons between countries and in particular regions of the world with large differences in diet show clear associations between a number of foods and cancer rates particularly hormone-dependent cancers [246, 247]. Similar comparisons within individual countries have however generally failed to replicate such associations; this may at least partly be due to the relatively small variations in diet within a country, which are not much greater than the errors in the methods for measuring diet [248]. The associations of cancer with some dietary components have been supported by considerable experimental evidence leading to large clinical trials with specific nutritional interventions and these have led to many prominent and very costly disappointments. A trial of the effects of a low fat, high fibre diet on the recurrence of colorectal adenomas in 1,429 subjects failed to demonstrate any effect [249] even after 8 years of follow-up [250]. Even more unsuccessful was supplementation with folic acid in 1,021 subjects, which increased the risk of recurrence of multiple or larger colorectal adenomas [251]. A large trial of β-carotene and vitamin E supplements to prevent lung cancer in 29,133 men was again unsuccessful with an outcome indicating that high-dose β-carotene supplementation increased the risk of lung cancer in smokers [252]. A randomised trial of selenium and vitamin E, alone or in combination, for the prevention of prostate cancer in 35,533 healthy men across 427 participating centers was discontinued early due to no evidence of benefit for either supplement [253].

Despite the failure to find a simple single dietary component that can generally reduce cancer, there is still considerable evidence that nutrition and metabolic status are of critical importance for the progression of the common epithelial cancers. Compared with the lack of success with food supplements in human populations, the most well established intervention for reducing cancer incidence in experimental animal models across a range of species is a general reduction in nutritional intake or calorie restriction [254]. Several mechanisms have been proposed for the protective effect, but calorie restriction consistently lowers the systemic levels of IGF-I and replacing the IGF-I with infusions in calorie restricted mice to prevent any reduction negated the benefits in terms of reduced tumour promotion [255, 256]. This implied that the reductions in IGF-I mediated the beneficial effect of dietary restriction. That reduced nutritional intake and reduced IGF-I activity act *via* a common pathway is also supported by the observation that dietary restriction fails to inhibit cancers induced by constitutive activation of signalling pathways downstream of the IGF-I receptor [257]. Direct evidence that calorie restriction reduces cancer in humans is lacking but there is some evidence that would be consistent with the effect being the same in humans. In Okinawa, Japan, up until the 1960s the diet was characterised by chronic low caloric intake relative to Japanese on the mainland with a diet containing approximately 10.9 % fewer calories than required for maintenance of body weight (according to Western criteria) and mortality rates for breast and prostate cancers were approximately half that of mainland Japan and mortality from colon cancer was 38 % lower in men and 60 % lower in women [258]. This observational study is consistent with a calorie restricted state reducing mortality in a Japanese population: a population where the rates of these cancers are already considerably lower than that in North America and western Europe.

In addition to nutritional interventions, interest has been growing in pharmaceutical interventions that target metabolic pathways. Following the many lines of evidence indicating that the IGFs are important for many cancers, virtually every pharmaceutical company invested heavily in developing drugs targeted at the IGF-I receptor. Despite the compelling preclinical evidence, the many trials for a range of cancers have largely been very disappointing; although all trials thus far have been on all patients without any attempt to select those that may be more responsive. There are several potential reasons for the failure of trials targeting the IGF-I receptor [259]: these include constitutive activation of the PI3K pathway due to the common mutations in PIK2CA or PTEN which render cells independent of upstream receptor activation; the absence of the receptor substrate, IRS-1, that leads to the receptor signalling for differentiation rather than proliferation or survival; the presence of alternatively spliced insulin receptors that respond to IGF-II which is frequently overexpressed due to imprinting defects and the activation of the ERK pathway due to overexpressed Ras or Raf again bypassing dependence on IGF-I receptor activation. The recent demonstration that an IGF binding protein-related protein IGFBP-rP1 binds directly to the IGF-I receptor and prevents ligand binding suggests an additional mechanism with tumours overexpressing this protein also becoming independent of IGF-I receptor and hence resistant to its targeting [260]. It is feasible that activation of the pathway is so essential for tumour progression, since growth will not occur without a signal indicating that a ‘fed-state’ is present, that by the time that such interventions are instigated most tumours have acquired an autonomous way to sustain the pathway activition.

One of the most effective preventative interventions has been the long-term use of daily aspirin, which inhibits the activity of cycloxygenase-2 (COX-2) and reduces prostaglandin activity. In a meta-analysis of eight trials the use of daily aspirin for 7.5 years or longer reduced mortality from all solid tumours by 31 % and death from gastrointestinal cancers by 59 % [261]. A recent analysis of regular aspirin use following diagnosis of colorectal cancer in two large cohorts reported that cancer-related death was reduced by 82 % in subjects with cancers characterised by mutations in the PIK3CA gene whereas in the much larger numbers of subjects with no mutations in PIK3CA there was absolutely no effect on mortality [262]. The large effect of aspirin on colorectal cancers has been suggested to be unlikely due to effects on PIK3CA tumours alone as these only account for 10–20 % of such cancers [263], but as discussed earlier, there are many other potential aberrations in this pathway in cancers with which COX-2 inhibitors may interact. These recent findings suggest that the efficacy of aspirin may depend on the activity of the PI3K pathway implying an interaction with metabolic regulation.

More direct evidence of the potential for pharmaceutical manipulation of metabolic pathways comes from the growing interest in the use of metformin, one of the most commonly used anti-diabetic drugs, for preventing or treating cancer. Surveillance studies of patients with diabetes have indicated lower rates of cancer in those treated with metformin [264]. Metformin has several potential mechanisms of action [265, 266] but one of the most well characterised is *via* activation of AMP-activated protein kinase (AMPK) an intracellular signalling enzyme that is normally activated when the ratio of AMP to ATP in the cell rises. This enzyme is therefore normally activated when energy stores, and hence ATP levels, are reduced and AMPK is activated by hormones such as adiponectin that signals when systemic adipose tissue stores of energy are depleted. Thus while insulin and the IGFs are systemic signals that indicate that the organism is in the ‘fed state’ and nutrients and energy are available to support growth: the activation of AMPK is the opposing signal indicating that circumstances are not appropriate to support growth. An effect of metformin reducing cancer progression would therefore be entirely consistent with all of the evidence regarding metabolism and the effects of insulin and IGFs on cancer progression. Tumours, just like tissues in early development, may only grow and develop when signals indicate metabolic conditions are appropriate. In addition to activating AMPK pharmacologically, a natural way to reduce ATP levels is *via* physical activity and exercise that burn off the energy. There has been a recent surge in interest in the role that physical activity and exercise may play as an exposure that could affect cancer progression and as a potential intervention [267, 268]. A Western lifestyle is characterised not only by nutritional excess but also by a very sedentary lifestyle.

## Obstacles

To further our understanding of the role of lifestyle and metabolism in the progression of these epithelial cancers and realise the potential to reverse the epidemics in Western societies, there are a number of obstacles that need to be overcome. The dogma that genetic damage is the rate-limiting step and that cancer is an intrinsic problem of the cancer cell has to be overcome. There has to be wider appreciation that most epithelial cancers in older humans arise from perfectly normal mutation rates and that most elderly humans harbour many latent occult tumours: it is clear that this is just normal aging. It follows that preventing the initiation of cancer may then actually require a cure for the aging process itself: an ambitious goal. However, as the vast majority of these lesions are indolent, the important and clinically relevant question is what makes these lesions progress to rapid growth and invasion in a few individuals. This is the step in carcinogenesis that is potentially modifiable. The emphasis should then shift to factors that promote progression and metastasis: features of the disease that makes cancer a life-threatening condition. This will require somewhat of a departure from the trend for evermore-closer gene-centric focus on autonomous cell regulation. A broader approach is needed to gain a more comprehensive understanding of the organism as a whole and the context in which cancer cells develop and progress. A more holistic approach to lifestyle, energy balance and diets is also required, rather than searching for a single dietary component or a magic bullet.

The use of cell culture models has evolved inadvertently in such a way that the context of these cells is vastly different from that of cells in solid tumours. Virtually, all cell culture work is undertaken with cells grown in media that contains grossly super-physiological levels of glucose with unlimited supply of oxygen, vitamins, multiple nutrients and either an excess of growth factors or undefined growth factors present in serum. None of these exposures even closely resembles the environment of cancer cells within human solid tumours and the metabolic status of the cells used in most laboratory studies will be nothing like that of cells in real tumours. We have shown that the response to chemotherapy agents of breast cancer cells cultured in euglycaemic conditions can be considerably different to that when the cells are grown in their conventional media that is very hyperglycaemic [201]. More work needs to be done with cells grown in conditions relevant to real human cancers in order to understand how their metabolic status affects their behaviour and response to therapies.

The use of appropriate animal models has to be re-evaluated which will require overcoming a number of dogmas that have spread through the research community regarding the importance of current models. Human cancers develop due to interactions between cancer cells and the host tissue. Context is critical (as described above): within tumours there is considerable genetic and epigenetic heterogeneity and the host tissues reside within human populations that constitute billions of different genotypes each adapted and modified by lifestyle and exposures. In stark contrast, the standard mouse model is a mouse strain that is genetically entirely homogeneous, every mouse is genetically identical, and the animals live a sedentary life with continuous access to food, within confined containers in standardised conditions with constant day-night cycles and hence no environmental stimuli. As a result, these animals are very inactive, relatively overweight, insulin resistant and live much shorter lives than ‘normal’ rodents living in their natural habitats that are considerably more active and feed intermittently [269]. The standard mouse model therefore examines the interaction between homogeneous cancer cells inoculated or transplanted into homogeneous mice that are in a single, very artificial, metabolic state. This is a very poor model of the infinitely variable genetic and phenotypic interactions that contribute to the development of human cancers [217]. The majority of human epithelial cancers are chronic diseases with estimates of between 12 and 25 years intervening between initiation and diagnosis [4]; in contrast most mouse models are initiated by the introduction of millions of cancer cells and have rapidly developing tumours with interventions started soon after tumour initiation. Intervening in a newly formed tumour may bare no relation to intervening in a tumour that has developed and evolved over decades. All of these factors are pragmatic, to expedite quick results, but all differ considerably from human cancers.

That mouse models are poor models of complex human diseases has been confirmed recently in a very comprehensive evaluation of the genomic response to inflammatory conditions: the genomic response in humans was very similar for inflammatory responses of different etiologies but the various mouse models correlated poorly with the human conditions and poorly with each other [270]. Indeed the conclusion from this large collaborative study was that the mouse models ability to mimic human inflammatory diseases was close to random. The problems encountered in getting that substantive work published illustrates the kind of barriers that have to be overcome when challenging ingrained dogmas. Like inflammatory diseases, human cancers are similarly complex diseases and yet most interventions are still initially validated in mouse models in which the mice are all of one single genotype (and often lacking an immune system). Humans die when their cancers metastasise whereas the drugs are tested against mouse xenograph tumours that very rarely metastasise. New drugs are tested in these models as mono-therapy or at most dual therapies whereas in humans most phase 3 trials normally involve the standard chemotherapy regimen with or without the new drug. An analysis of 213 phase 1 clinical trials between 1991 and 2001 involving 6,474 cancer patients found the overall objective clinical response rate was just 3.8 % with a toxic death rate of 0.54 % [271]. Almost all of the drugs tested would have been initially validated in mouse subcutaneous xenograft models, and if these combined trials were viewed as an experiment to ask whether these models mimicked the response in humans then, with 96.2 % of humans failing to respond as in the mouse models, the conclusion would clearly be negative. Recently, much better mouse models have been developed with more orthotopic cancer models and transgenic models in which genes can be switched on or off to initiate endogenous tumours in mice with intact immune systems; but there is still a lack of evidence that these new models will better predict the response of human cancers [217]. Most genetically engineered mouse models result in rapidly developing tumours with one single genotype in mice that are also genetically homogeneous: such models test the interaction of a single cancer genome with a single host genome. It is now clear that human tumours are extremely heterogeneous even within an individual and these tumours occur in individuals with infinitely variable genotypes and extrapolating findings from the genetically engineered mice can only give a very limited picture of the complexity of the human disease. A better understanding of the human disease will require a shift from model systems to more translational clinical research.

The variability, lack of rigour and lack of reproducibility of preclinical cancer research was highlighted by a recent report from the oncology laboratory of a prominent biotechnology firm where they tried to reproduce the findings from 53 landmark publications and found that the findings of only 6 of these studies could be confirmed [272].

If dietary and lifestyle exposures contribute considerably to the progression of many epithelial cancers then a better understanding could lead to more effective prevention and treatments. There will then undoubtedly be obstacles in terms of acceptability and compliance: it is likely that severe calorie restriction may work—but it would not be very acceptable to many and difficult to attain good compliance as a public health measure. Our first priority must however be to provide the evidence and then people will at least have the choice of whether to act on that evidence.

## Summary: the soil and the systemic milieu

Mutations to oncogenes and tumour suppressor genes occur in millions of cells in all of us throughout our lifespan. The accumulation of these mutations are undoubtedly essential to cancer initiation, however they do not appear to be sufficient for the clinical progresson of most epithelial cancers. Mutations occur very frequently and even mutations in oncogenes and tumour suppressor genes very rarely ever result in the initiation of a neoplastic growth and very few of these neoplastic lesions ever progresses to form a tumour of clinical significance. Although the seeds are plentiful, it is clear that they only germinate if the soil is right: the internal milieu is set not only by local tissue architecture but also by the availability of metabolic substrates and the levels of hormones, growth factors and cytokines that regulate these. Metabolic control is not just a hallmark of cancer it is even more fundamental, it is an axiom of life itself: every biological function is dependent on the availability of the necessary substrates and the generation of energy and therefore metabolism has to be regulated accordingly. Cells must change behaviour to form a tumour; most of the changes involved could just be due to inappropriate activation of programs that are normally only active in early development or during wound healing. The necessary resetting of cell metabolism may be a direct consequence of this reactivated program. The autonomous cell metabolism controls that evolved in single cells have however been superceded in complex metazoans by systemic regulators that ensure that all tissue functions are synchronised to the metabolic status of the organism as a community of cells. While malignant cells are in many ways antisocial and no longer work for the good of the general cell community, the accumulating evidence indicates that systemic metabolic conditions still greatly influence the ability of neoplastic epithelial cells to transform and adopt a malignant phenotype [273].

As we age, the genetic damage accumulates but the soil also changes. Age is the strongest risk factor for the common epithelial cancers and although many of these cancers are dependent on sex steroids they most commonly occur at a time in the lifespan when the levels of sex steroids are declining. As described above, IGF-I is heavily implicated for also playing an important role in these common epithelial cancers, but the levels of IGF-I also decline with age and are relatively low at the age when these cancers commonly occur. When rhabdomyosarcoma cells were inoculated into rats of different ages the number of resulting lung tumours was greatest in the youngest rats and correlated positively with the IGF-I-activity in the lung [274]. Some clinical evidence that age-related changes in the internal milieu affects tumour growth have also been reported; for example, the local recurrence of breast cancer in a long-term follow-up after partial mastectomy was found to be considerably higher in women younger than 46 years compared with women older than 65 and women younger than 35 had an increased risk of distant metastases [275]. It is possible that the age-related decline in levels of hormones may confer some protection against the development of cancers due to the inevitable accumulating genetic damage. This protection against cancer afforded by a soil that becomes increasingly less fertile for tumour development may be dependent on age-related changes in hormones but these hormones are also heavily influenced by lifestyle. Our current lifestyles are characterised by high-energy intake and low energy-expenditure working together to create a large energy imbalance. Our sedentary living patterns are exacerbated by a combination of automated transport and increasingly desk-based work and television/online recreational activities. These decreases in energy-expenditure have occurred at the same time as dramatic changes in nutrition with diets comprised of mainly energy-dense processed foods. Our current lifestyles entail an energy-imbalance that would have been extremely rare throughout the many millenniums of evolution up until the very last few decades and hence the design has not evolved to adapt to such conditions and the consequences of such a metabolic imbalance are clearly evident from the epidemics of obesity and diabetes. This metabolic imbalance has however also dramatically altered the soil in which many latent occult cancers naturally accumulate as we age (Fig. 5).

## Conclusions

In combination, these different lines of evidence not only indicate the importance of metabolism for the common epithelial cancers and the major role played by lifestyle and environmental exposures, but they also importantly suggest that most cancers are in principle avoidable even when there is a high genetic susceptibility. The seeds for cancers are plentiful and accumulate naturally with aging; they however need the right soil and the soil needs to be fertile and this is strongly influenced by lifestyle. With a better understanding of the lifestyle and environmental exposures that influence risk and progression, effective prevention should therefore be feasible. The scale of potential benefit can be assessed by the protection from the cancers that are common in Westernised countries that was afforded by the Japanese lifestyle as in the 1950s: that the protection was due to lifestyle and not genes has been confirmed by many studies including evidence from the rapid changes in Japan over the following years and also evidence from studies of migrants. When compared with the USA mortality from breast cancer was 92 % lower, from prostate cancer 86 % lower and from colorectal cancer 78 % lower in men and 81 % lower in women [144]. With such a large potential to reduce the burden of cancer throughout the developed world, it is remarkable that research in this area has received relatively so little emphasis. The re-emergence, or re-discovery, of metabolism as a central issue in cancer research could take us further to realising this potential.
